# Dysregulation of Lipid Metabolism in Macrophages Is Responsible for Severe Endotoxin Tolerance in FcgRIIB-Deficient Lupus Mice

**DOI:** 10.3389/fimmu.2020.00959

**Published:** 2020-06-09

**Authors:** Thiranut Jaroonwitchawan, Peerapat Visitchanakun, Phi Cong Dang, Patcharee Ritprajak, Tanapat Palaga, Asada Leelahavanichkul

**Affiliations:** ^1^Department of Microbiology, Faculty of Medicine, Chulalongkorn University, Bangkok, Thailand; ^2^Department of Microbiology, Faculty of Dentistry, Chulalongkorn University, Bangkok, Thailand; ^3^Department of Microbiology, Faculty of Science, Chulalongkorn University, Bangkok, Thailand; ^4^Translational Research in Inflammation and Immunology Research Unit (TRIRU), Department of Microbiology, Chulalongkorn University, Bangkok, Thailand

**Keywords:** endotoxin tolerance, Fc gamma receptor IIb, macrophage, lupus, AMPK, phosphatidylethanolamine

## Abstract

FcgRIIB dysfunction is commonly found in patients with lupus, especially in Asia. LPS-tolerance is prominent in FcgRIIB–/– lupus mice. LPS-tolerant macrophages demonstrate cell energy depletion, which might affect lipid metabolism. Therefore, to explore lipid metabolism, LPS-tolerance was induced twice by LPS administration in macrophages and in mice. LPS-tolerant FcgRIIB–/– macrophages demonstrated lesser mitochondrial DNA (mtDNA), more severe ATP depletion, lower cytokine production, and higher lipid accumulation (oil red O staining) compared to LPS-tolerant WT cells. Mass-spectrometry-based lipidomic analysis demonstrated a higher abundance of phosphatidylethanolamine (PE) phospholipid in LPS-tolerant FcgRIIB–/– macrophages than WT cells. This was at least in part due to the lower expression of phosphatidylethanolamine N-methyltransferase (*pemt*), an enzyme that converts PE to phosphatidylcholine (PC). Aminoimidazole-4-carboxamide ribonucleotide (AICAR), a *pemt* inhibitor, worsens LPS-tolerance in WT macrophages and supports the impact of *pemt* upon LPS-tolerant FcgRIIB–/– macrophages. Additionally, phosphorylated AMP-activated protein kinase (AMPK-p), a molecule for ATP-restoration associated with *pemt*, and phosphorylated acetyl CoA carboxylase, a downstream signaling of AMPK-p, were higher in LPS-tolerant FcgRIIB–/– macrophages than WT. Furthermore, Compound C, an AMPK inhibitor, attenuated LPS-tolerance in both FcgRIIB–/– macrophages and mice. Taken together, the intense decrease in cytokine production after the second LPS stimulation (LPS-tolerance) in FcgRIIB–/– macrophages was possibly due to the impact of an immense cytokine synthesis after the first dose of LPS. This includes using up PEMT, an enzyme of phospholipid synthesis during cytokine production, and AMPK-p induction in response to profound ATP-depletion. Therefore, the manipulation of the AMPK/PEMT axis provides a novel therapeutic candidate for the treatment of severe LPS-tolerance in lupus.

## Introduction

One cause of systemic lupus erythematosus (SLE) is the functional defect in Fc gamma receptor IIB (FcgRIIB). This is the only inhibitory receptor among members of the FcgR family, especially in Asian population (1–3). In a mouse model of lupus, FcgRIIB–/– mice exhibited effective microbial control against several micro-organisms due to enhanced immune responses from inhibitory signaling loss (2, 4, 5). However, immune responses in these mice showed an extremely exhausted phenotype after repeated stimulation, as demonstrated by the intense reduction of cytokine production after repeated LPS stimulation, often referred to as “LPS-tolerance” (6, 7). Inadequate cytokine production due to prominent LPS-tolerance in FcgRIIB–/– lupus mice enhances sepsis susceptibility (6, 7). This is similar to a secondary infection after immune exhaustion or immune paralysis (8–10). As such, LPS-tolerance might be responsible for the increased infection susceptibility in lupus patients (11, 12).

Interestingly, it has been reported that LPS-tolerance is possible in active lupus because of spontaneous endotoxemia both in patients and in mice (13, 14). This could possibly lead to persistent LPS stimulation and LPS-tolerance (15–18). While LPS-tolerance protects the host from lethal doses of LPS by dampening cytokine responses, cytokine levels in LPS-tolerance are too low for organism control (6). Although TLR4 signaling, microRNA, epigenetic alteration (16), and cellular metabolism (18) have been proposed as the mechanisms of LPS-tolerance, evidence is still inconclusive, and studies of LPS-tolerance in lupus are still lacking. Due to the hyper-responsiveness to LPS stimulation in FcgRIIB–/– macrophages (6, 7), prominent LPS-tolerance of FcgRIIB–/– cells might result from profound energy insufficiency (18–20) or post-translational modification (7, 21). Fatty acids are the source of β-oxidation, which is a catabolic process that converts fatty acids into acetyl-CoA for Krebs cycle in mitochondria of eukaryotic cells (22). Therefore, further lipid exploration in LPS-tolerant macrophages is interesting because: (1) β-oxidation is important for mitochondria (22); (2) intracellular lipids are responsible for diverse cell functions, such as phagocytosis, cytokine production, and mitochondrial function (23, 24); and (3) increased lipid accumulation in activated macrophages has been documented (25). The comparison between bone marrow-derived macrophages from FcgRIIB–/– and WT with either single or sequential LPS stimulation has led to the identification of lipid metabolism as one of the pathways that regulates LPS-tolerance and depresses cytokine production in LPS-tolerant FcgRIIB–/– macrophages.

## Materials and Methods

### Animal and Endotoxin-Tolerance Mouse Model

Animal study protocols were approved by the Faculty of Medicine, Chulalongkorn University, following the National Institutes of Health (NIH) criteria. FcgRIIB–/– mice (on C57BL/6 background) were provided by Dr. Sylvia M. Bolland (NIAID, NIH, Maryland, USA). Other mice were purchased from the National Laboratory Animal Center, Nakornpathom, Thailand. Eight-week-old female C57BL/6J mice were used in all experiments. The endotoxin-tolerance model was performed by intra-peritoneal administration of endotoxin (LPS) from *Escherichia coli* 026:B6 (Sigma-Aldrich, St. Louis, MO, USA) in two separate doses at 0.8 and 4 mg/kg with 5-day rest between doses (6). Then, blood was collected through tail vein nicking at specific time-points after the second dose of LPS to measure serum cytokines (TNF-α, IL-6, and IL-10) by ELISA (Bioplex, Bio-RAD, CA, USA). In addition, Compound C, an inhibitor against AMP activated protein kinase (AMPK), was tested in mice. Accordingly, 1 mg/kg of Compound C (Dorsomorphin, Sigma-Aldrich) was intra-peritoneally administered following a previous publication (26) together with the second dose of LPS in the mouse model.

### Bone Marrow-Derived Macrophages, Endotoxin Stimulation Protocol, and Manipulation

Activation of bone marrow (BM)-derived macrophages from progenitor cells in mice femur with L929-conditioned media was supported by flow cytometry by anti-F4/80 and anti-CD11c antibody (BioLegend, San Diego, CA, USA) before use (6, 7). Then, endotoxin (LPS) *Escherichia coli* 026:B6 (Sigma-Aldrich) at 100 ng/ml in 100 μl/well was used to activate macrophages (1 × 10^5^ cells/well) with two protocols, including single incubation and 2-sequential LPS stimulation (6, 7). For single LPS stimulation (N/100), culture media without endotoxin was used for 24h then washed with phosphate buffer solution (PBS) and filled with LPS. For sequential LPS stimulations (100/100), LPS was incubated for 24 h, washed, and refilled with the same dose of LPS. In the control group (N/N), culture media without endotoxin was used before and after washing. Culture supernatant was collected at indicated time-points after the second incubation and stored at −80°C until cytokine analysis by ELISA (Thermo-Fisher Scientific, Waltham, MS, USA). In addition, aminoimidazole-4-carboxamide ribonucleotide (AICAR), an inhibitor of phosphatidylethanolamine N-methyltransferase (*pemt*), and Compound C, an AMPK inhibitor, were used to test the impact on LPS-tolerance. AICAR (50, 100, or 200 μM) or Compound C (5 μM) (Sigma-Aldrich) was incubated in macrophages together with the second dose of LPS in sequential LPS protocol (100/100) before supernatant cytokine determination.

### Macrophage Phagocytosis

Macrophage phagocytosis was measured by incubation of zymosan conjugated with 40 kDa fluorescein isothiocyanate dextran (FITC-dextran) (Sigma-Aldrich) at 200 μg/ml in 1 x 10^5^ cells/ well for 1 h at 37°C in 5% CO2 following a previous protocol (27). Then, the extracellular fluorescence was washed out by PBS and quenched by Trypan blue solution. After that, residual adherent macrophages were fixed with 4% paraformaldehyde and stained with Hoechst 33342 nuclear stain (Molecular Probe, Eugene, OR, USA). Cells were explored by an Olympus IX81 inverted fluorescence microscope. The ratio of fluorescence intensity of FITC-dextran, normalized by the number of nuclear staining with a Varioskan Flash microplate reader, was used to represent phagocytosis activity.

### Mitochondria Staining and Extracellular Flux Analysis

LPS-tolerance might be associated with cell-energy adaptation. Therefore, several parameters of mitochondria were explored. As such, 200 nM of Mitotracker Red CMxROS (Molecular probe) was added to each well and incubated at 37°C for 15 min before removal. Then, cells were fixed with cold methanol at −20°C for 15 min, washed twice with PBS, and photographed by an IX81 inverted microscope (Olympus, Tokyo, Japan). Energy metabolism profiles with estimation of glycolysis were performed and assessments of mitochondrial oxidative phosphorylation with extracellular acidification rate (ECAR) and oxygen consumption rate (OCR) were carried out by Seahorse XFp Analyzers (Agilent, Santa Clara, CA, USA) on macrophages at 1 × 10^5^ cells/ well by Seahorse Wave 2.6 software as previously described (21).

### Quantitative Real Time PCR (qRT-PCR) for Mitochondrial Genome (mtDNA), Cytokines, and Lipid Metabolism Enzymes

For mitochondrial DNA (mtDNA) detection, total DNA was extracted by FavorPrep™ Tissue Genomic DNA Extraction assay (Favorgen, Ping-Tung, Taiwan) before analysis by real time RT-PCR with the following sequences encoded for mtDNA (mmito-1); forward: 5′-CGTACACCCTCTAACCTAGAGAAGG-3′, reverse: 5′-GGTTTTAAGTCTTACGCAATTTCC-3′; compared to the following house-keeping sequences of β2 microglobulin (β*2*M); and forward: 5′-GGACAGTGGGTAGGGAACTG-3′, reverse: 5′-GGACAGTGGGTAGGGAACTG-3′ (28). Then, mtDNA relative to β2M was analyzed by the comparative threshold cycle (ΔCt) method.

For cytokines and enzymes in lipid metabolism, total RNA was extracted by an RNeasy mini kit (Qiagen, Albertslund, Denmark). Then, RNA (200 ng) was converted into cDNA in 20 μl of reaction mix by RevertAid First Strand cDNA synthesis Kit (ReproTech, Oldwick, NJ, USA). Primers used for these experiments are demonstrated in Table 1. Gene expression relative to β*2M* were analyzed by 2−ΔΔCT method, and fold change between the interested conditions and untreated WT macrophage control (N/N) were demonstrated. Measurements of transcript levels were performed with mastermix 1xKAPA fast SYBR Green (Kapa Biosystems, Wilmington, MA, USA). Real time RT-PCR was performed by a QuantStudio® 6 Real-Time PCR system (Applied Biosystems, Life Technology Corporation, CA, USA) with a final volume reaction of 10 μL containing 0.3 μmol/L of each forward and reverse primer. Mastermix 1xKAPA fast SYBR Green (Kapa Biosystems, Wilmington, MA, USA) and 2 μL of DNA template were used.

**Table 1:** A list of primers.

**Primers**	**Forward**	**Reverse**
Tumor necrosis factor α (TNF-α)	5′-CCTCACACTCAGATCATCTTCTC-3′	5′-AGATCCATGCCG TTGGCCAG-3′
Interleukin-10 (IL-10)	5′-GCTCTTACTGACTGGCATGAG-3′	5′-CGCAGCTCTAGGAGCATGTG-3′
Phosphatidylethanolamine N-methyltransferase (pemt)	5′-TGGCTGCTGGGTTACATGG-3′	5′-GCTTCCGAGTTCTCTGCTCC-3′
Choline/ethanolamine phosphotransferase (cept1)	5′-GCTCACTCTAATCATCACTA-3′	5′-CCTGTTGTCCTTAATATGTTC-3′
Ethanolamine kinase (ek2)	5′-AGCATCCTCTTCCACTTCTC-3′	5′-TTCCGCCATTCAGTTCCA-3′
Phosphatidylserine decarboxylase (psd)	5′-TGAGGACAATGACTAATGATG-3′	5′- ACCAGACAAGCCAGTAAT-3′
Phosphocholine cytidylyltransferase (pct1)	5′-CTTCTATCAGATTGACAGT-3′	5′-CTAATTCCTTGGCTTCTT-3′
β2 microglobulin (β2M)	5′-TTCTGGTGCTTGTCTCACTGA-3′	5′-CAGTATGTTCGGCTTCCCATTC-3′

### Reactive Oxygen Species (ROS), Adenosine Triphosphate (ATP) Measurement, and Cell Viability Test

Cellular total ROS production was determined by fluorescent dye, Dihydroethidium (DHE), according to the manufacturer's protocol. Briefly, 20 μM of DHE (Sigma-Aldrich) was incubated for 20 min at 37°C before DHE measurement at the indicated time-points. Fluorescence readings were analyzed at 520 nm by a Varioskan Flash microplate reader as presented by fluorescence arbitrary unit (a.u.). Cellular ATP content was identified by incubation with the substrate from Luminescent ATP Detection Assay (Abcam, San Francisco, CA, USA) for 15 min in room temperature before analysis with a Varioskan Flash microplate reader (Thermo-Fisher Scientific) following the manufacturer's instruction. In addition, cell viability was analyzed by tetrazolium dye 3-(4, 5-dimethylthiazol-2-yl)-2, 5-diphenyltetrazolium (MTT) solution (Thermo Fisher Scientific) according to the manufacturer's protocol. In short, macrophages at 1 × 10^5^ cells/well were incubated with 0.5 mg/mL of MTT solution for 2 h at 37°C in the dark. Then, MTT was removed and diluted with Dimethyl sulfoxide (DMSO; Thermo Fisher Scientific) before measurement with a Varioskan Flash microplate reader with absorbance at 570 nm.

### Total Lipid Staining by Oil Red O Dye and Fluorescent-Labeled Phospholipid Uptake

Cells were washed twice with PBS before staining with 0.3% Oil Red O solution (Sigma-Aldrich) for 10 min, washed with PBS, fixed with 4% paraformaldehyde for 15 min at room temperature, and washed again with PBS. Stained cells were evaluated under a microscope with 10 random fields from each well. Intensity was evaluated by ImageJ (NIH, Bethesda, MD, USA). Although cellular lipid accumulation might be due to increased lipid synthesis or enhanced uptake, and only lipid uptake was tested due to technical limitations of lipid synthesis (29, 30). Fluorescent phospholipid analogs (Avanti Polar Lipids, Inc., AL, USA), including lipid analogs 1-acyl-2-[12-(7-nitro-2,1,3-benzoxadiazol-4-yl) amino]dodecanoyl]-sn-glycero-3- phosphocholine (NBD-PC) and 1,2-dipalmitoyl-sn-glycero-3-phosphoethanolamine-N-(lissamine rhodamine B sulfonyl) (Rhodamine liss PE), were prepared in 100% ethanol for the uptake assay following a published protocol (31). Briefly, 20 μM of phospholipid analogs were added to macrophages in a 96-well plate. At indicated time-points, cells were washed twice, and PBS (50 μl) was added to each well. Fluorescence of NBD-PC and Rhodamine-liss PE taken up by cells was measured with Varioskan Flash microplate reader (Thermo-Fisher Scientific). Phospholipid uptake was calculated by ratio between intensity of phospholipid reporter and number of nuclei as stained by Hoechst 33342 (Molecular Probe).

### Lipid Extraction and LC-MS Data Analysis

A metabolomics protocol for sample preparation for adherent cells was followed (32). In short, culture media of 2 × 10^6^ cells/ well of macrophages were removed and placed on dry ice before 4 ml of 80% (vol/vol) methanol (cooled to −80°C) was added. Plates were then incubated in −80°C for 20 min before scraping to separate the cells. Samples were transferred to 15 ml conical tube and centrifuged at 14,000 g for 10 min at 4°C before removal of the metabolite-containing supernatant to a new conical tube on dry ice. Subsequently, lipid components in the pellet were dissolved again with 80% (vol/vol) methanol according to previously mentioned procedures. The metabolite-containing supernatant from the pellet was combined with the previous supernatant before being concentrated with the total lipids from cell lysate by speed vacuum at −56°C for 16h to evaporate MeOH and then stored at −80°C before further analysis. Total lipids were reconstituted in 100 μl of 50% MeOH in water, MeOH:H2O 1:1 (v/v), and analyzed by an untargeted approach based on liquid chromatography coupled with an electrospray-ionization LC ESI-MS using micrOTOF-Q II (Bruker Daltonics, USA) with a chromatography system following the previous condition (33, 34). In short, LC/MS/MS Q-TOF with LC separation on a C18 column (Agilent, Santa Clara, CA, USA) and mobile phases consisting of ultrapure water and acetonitrile containing 0.1% (v/v) formic acid were used. Data analysis was performed by Profile analysis software (Bruker Daltonics, USA) for LC/MS data. The difference between data sets was determined by using unpaired two-tailed Student's *t*-tests. The Lipidomics Gateway (http://www.lipidmaps.org) was used to identify lipids based on major fragment ions of the MS spectrum after manual monoisotope selection. LC/MS data was subjected to Metaboanalyse 3.0 (https://www.metaboanalyst.ca) for principal cluster analysis (PCA), Volcano plots, and enrichment pathway analysis. The enrichment pathway analysis was performed as a Metabolite Set Enrichment Analysis by Metaboanalyse 3.0. This method was used to identify biologically meaningful patterns that were significantly enriched in quantitative metabolomic data. The intensity between data sets was compared using unpaired two-tailed Student's t-tests with profile analysis software. Only unknown metabolites with *p* < 0.01 were considered for further analysis. Out of 1,765 lipids, there were 187 selected unknown metabolites that were then identified by lipidomic map database with search parameters including Ion adducts: “M+H”, “M+H-H2O”, “M+Na”, “M+NH4”, “M+K”, and Mass Tolerance (m/z): +/– 0.05.

### Western Blot

Western blot analysis was used to explore the abundance of AMPK and phosphorylated-AMPK (AMPK-p) and phosphorylated-acetyl-CoA carboxylase (ACC-p), a downstream signaling of AMPK-p, in LPS-tolerant macrophages following the standardized procedure (7, 21). In brief, stimulated macrophages were pelleted, washed with PBS, and lysed in RIPA lysis buffer with a protease/phosphatase inhibitor (Thermo-Scientific, Rockford, IL, USA). The samples were homogenized and protein quantification was performed by Bicinchoninic acid assay (BCA) (Pierce BCA Protein Assay, Thermo Scientific) and then separated in 10% sodium dodecyl sulfate (SDS) polyacrylamide gel before transferring into a nitrocellulose membrane. Subsequently, the preparation was incubated with primary antibodies for AMPK, AMPK-p, and ACC-p (Abcam, Cambridge, MA, USA), probed with the proper anti-IgG horseradish peroxidase (HRP)-conjugated secondary antibody (Santa Cruz Biotechnology), and detected by an enhanced chemiluminescence rapid step chemiluminescence detection system (Thermo-Scientific). Rabbit monoclonal antibody of glyceraldehyde 3-phosphate dehydrogenase (GAPDH) (Abcam) was used as a housekeeping control.

### Statistical Analysis

All statistical analyses were performed with GraphPad prism 5.0 (GraphPad Software, Inc., San Diego, CA). *In vitro* data were based on triplicate independent experiments and represented by mean ± standard error (SE). A *p* value < 0.05 was considered statistically significant. Student's t-test or one-way analysis of variance (ANOVA) with Tukey's comparison test was used for the analysis of experiments with two and more than two groups, respectively.

## Results

### Characteristics of FcgRIIB–/– Macrophages After Single or Sequential LPS Stimulation

**Figure 1:**
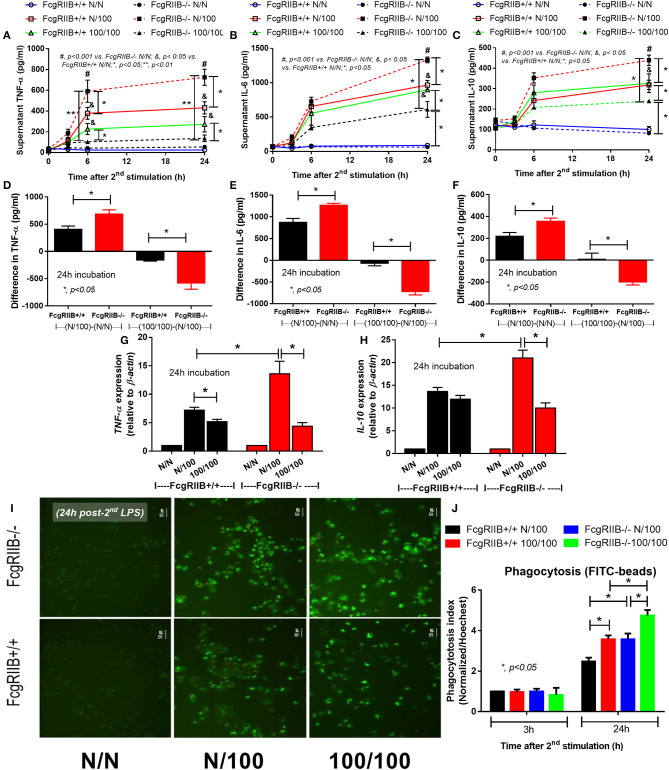
Characteristics of wild-type (FcgRIIB+/+) macrophages and FcgRIIB–/– cells after single (N/100) or sequential (100/100) LPS stimulation vs. control (N/N) as determined by time-course of supernatant cytokines (TNF-α, IL-6, and IL-10) **(A–C)**, the difference of cytokine level between single LPS stimulation vs. baseline [(N/100)–N/N)] and between single LPS stimulation vs. LPS tolerance [(100/100)–(N/100)] **(D–F)** and gene expression of *TNF-*α and *IL-10* **(G,H)** are demonstrated. Phagocytosis function of these experimental groups evaluated by the uptake of FITC-Zymoxan beads in the representative figures **(I)** and phagocytosis score **(J)** are also shown (Independent triplicate experiments were performed; *p < 0.05; **p < 0.01).

Both single (N/100) and sequential (100/100) LPS stimulation induced TNF-α and IL-6 (Figures 1A,B) in WT and in FcgRIIB–/– macrophages. However, cytokine levels in FcgRIIB–/– cells with single LPS stimulation were significantly higher than those in WT macrophages. In contrast, cytokine levels in FcgRIIB–/– cells with sequential LPS stimulation (LPS-tolerance) were lower than WT cells (Figures 1A–C). Higher responses against single LPS stimulation and more depressed cytokine production during LPS-tolerance of FcgRIIB–/– cells were also demonstrated by the difference in cytokine levels between single LPS and control (N/100-N/N) and between sequential LPS vs. single LPS [(100/100)–(N/100)] (Figures 1D–F). In parallel, gene expression of a pro-inflammatory cytokine (*TNF-*α) and anti-inflammatory cytokine (*IL-10*) was higher in single LPS-stimulated FcgRIIB–/– macrophages than WT cells (Figures 1G,H). In LPS-tolerant (100/100) WT macrophages, *TNF-*α expression, but not *IL-10* expression, was lower than single LPS stimulation. The expression of both genes in LPS-tolerant FcgRIIB–/– cells was lower than single LPS activation (Figures 1G,H). These data support that the inhibitory signaling loss of FcgRIIB–/– macrophages induces hyper-responsiveness after single LPS stimulation and is followed by intense unresponsiveness to the second dose of LPS (LPS-tolerance) (6). In addition, LPS-tolerance was also accompanied by enhanced phagocytosis activity in both WT and FcgRIIB–/– macrophages. There was higher activity in FcgRIIB–/– cells (Figures 1I,J) at 24 h after the second LPS stimulation, which supports previous findings (35).

### Immense Reduction of Mitochondria Quantity and ATP Production in LPS-Tolerant FcgRIIB–/– Macrophages

**Figure 2:**
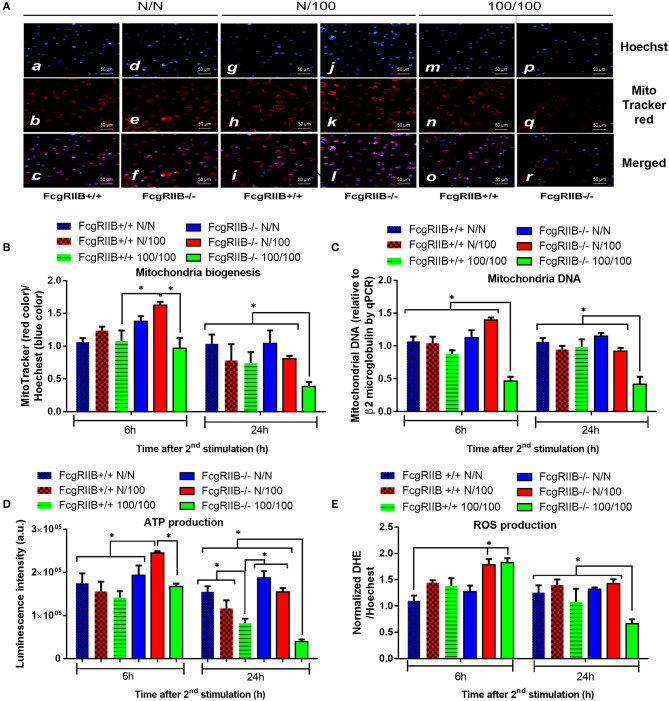
The evaluation of cell energy of wild-type (FcgRIIB+/+) macrophages and FcgRIIB–/– cells after single (N/100) or sequential (100/100) LPS stimulation vs. control (N/N) as determined by (1) mitochondrial assessment with Mitotracker Red as presented in representative figures from 6 h post-stimulation **(A)** and quantitative score **(B)**, (2) semi-quantitative expression of mitochondrial DNA content (mtDNA) in relative to β2 microglobulin (β2M) gene by qRT-PCR **(C)**, and (3) luminescence intensity of cellular ATP production **(D)** are demonstrated. Total cellular reactive oxygen species (ROS) as evaluated by Dihydroethidium (DHE) assay **(E)** are also shown (Independent triplicate experiments were performed; *p < 0.05).

**Figure 3:**
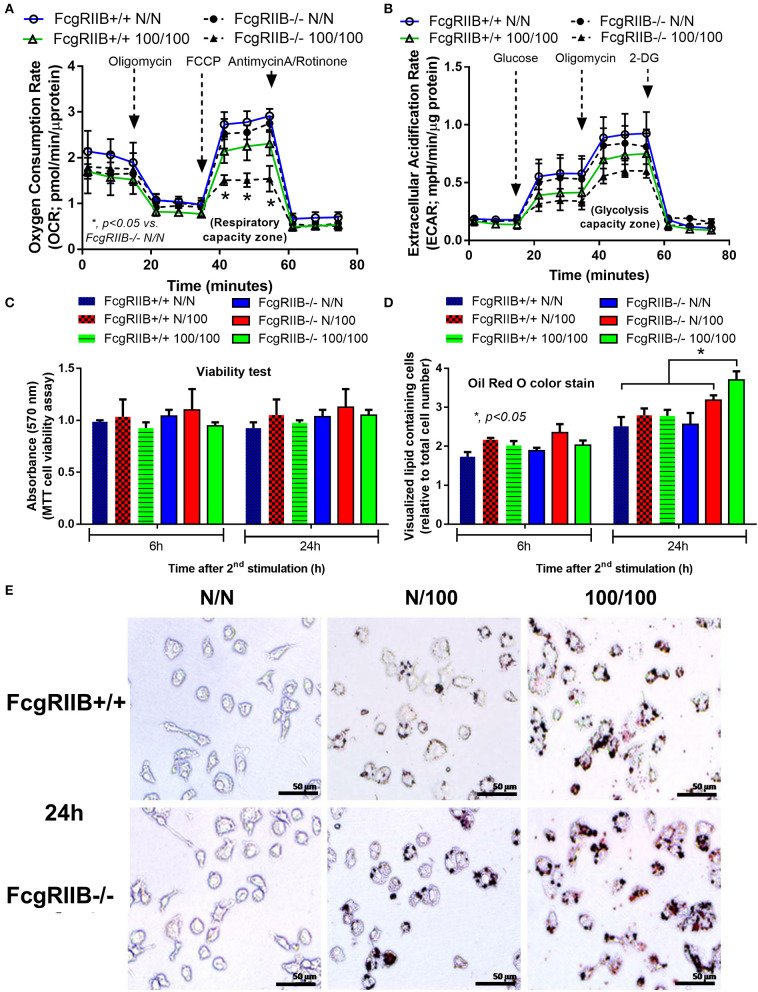
Extracellular flux analysis pattern of macrophages with LPS-tolerance (100/100) or culture media control (N/N) in FcgRIIB–/– and wild-type (FcgRIIB+/+) groups as evaluated by oxygen consumption rate of mitochondrial stress test for mitochondrial pathway analysis **(A)** and extracellular acidification rate of glucose stress test for glycolysis pathway analysis **(B)** at 6 h after the second dose of LPS or control culture media are demonstrated. The evaluation on macrophages of wild-type (FcgRIIB+/+) and FcgRIIB–/– cells after the single (N/100) or the sequential (100/100) LPS stimulation vs. control (N/N) in the viability assay analyzed by tetrazolium dye 3-(4, 5-dimethylthiazol-2-yl)-2,5-diphenyltetrazolium (MTT) solution **(C)**, the number of lipid droplets intensity determined by ImageJ software from Oil Red O staining **(D)**, and the representative Oil Red O staining from macrophages in each condition **(E)** are demonstrated (Independent triplicate experiments were performed for all figures; FCCP, Carbonyl cyanide-4-(trifluoromethoxy)-phenylhydrazone; 2-DG, 2-Deoxy-d-glucose).

Profound cytokine defect observed in LPS-tolerant macrophages is partially caused by cell-energy depletion and is often referred to as “inflammatory bioenergetics responses” (36). Therefore, cell energy was explored in FcgRIIB–/– macrophages. When comparing FcgRIIB–/– macrophages with WT cells, LPS-tolerance (100/100) reduced mitochondrial quantification and, as evaluated by MitoTracker, mitochondrial DNA (mtDNA). It also reduced ATP production and total cellular reactive oxygen species (ROS) (Figures 2A–E), especially at 24 h post-stimulation. In addition, cell energy evaluation by extra-cellular flux analysis demonstrated lower respiratory capacity (Figure 3A) and a tendency of lower glycolysis capacity (Figure 3B) in LPS-tolerant FcgRIIB–/– macrophages compared with control FcgRIIB–/– cells with the non-different cell viability (Figure 3C). Meanwhile, LPS-tolerance in WT macrophages showed a tendency of low mitochondria and glycolysis capacity compared with WT control but did not reach a significant value (Figures 3A,B). This implies severe energy insufficiency in LPS-tolerant FcgRIIB–/– cells and the depletion in cell-energy might be associated with low cytokine production (Figures 1A–D) in LPS-tolerant FcgRIIB–/– macrophages. The number of mitochondria, mtDNA, ATP production, and total cellular ROS in single LPS-stimulated FcgRIIB–/– macrophages (N/100) at 24 h (Figure 2) and extracellular flux analysis (Figures 3A,B) were not in the highest levels among all groups despite demonstrating the highest cytokine levels (Figures 1A–C). This may suggest less correlation between cell-energy status and cytokine production in single LPS stimulation.

### Increased Lipid Droplet Accumulation and a Global Shift in Glycerophospholipids Profile in FcgRIIB–/– Macrophages With LPS-Tolerance

**Figure 4:**
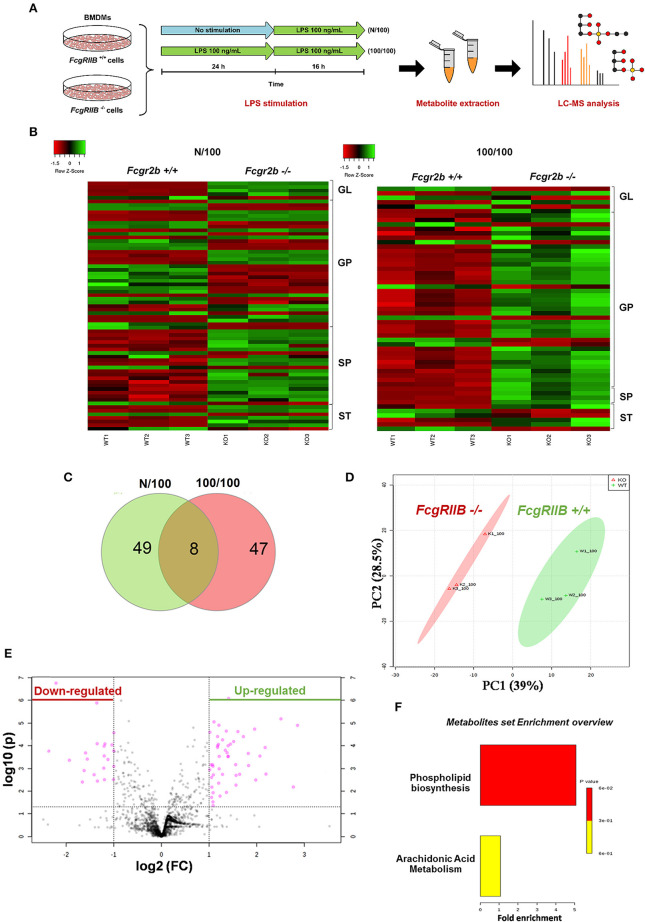
Schematic of experimental design for mass-spectrometry (LC-MS) based lipidomic analysis from bone marrow derived macrophages (BMDM) of FcgRIIB–/– and wild-type (FcgRIIB+/+) with single (N/100) and sequential (100/100) LPS stimulation **(A)** is demonstrated. Lipidomic analysis presented by hierarchical clustering heat-map profiling groups of lipid derivatives identified as glycerolipid (GL), glycerophospholipid (GP), sphingosine (SP), and sterol (ST) between wild-type (FcgRIIB+/+) and FcgRIIB–/– macrophages after LPS stimulation with the green and red color indicated up and downregulated lipid derivatives, respectively, **(B)** are shown. Venn diagram indicating the number of lipid derivatives in FcgRIIB–/– macrophages with N/100 or 100/100 LPS stimulation **(C)**, principal cluster analysis (PCA) in principle component 1 (PC1) identifying variance between individual groups from wild-type (WT1-3) vs. FcgRIIB–/– (K1-3) and in principle component 2 (PC2) indicating variance between replicate experiments **(D)**, Volcano plot of lipid derivatives quantification from sequential LPS stimulation (100/100) in wild-type vs. FcgRIIB–/– cells summarizing up- and downregulated lipid derivatives based on the adjusted log2 fold change **(E)**, and enrichment pathway analysis **(F)** are also indicated.

Severely reduced mitochondria biogenesis and decreased ATP in LPS-tolerant FcgRIIB–/– macrophages (Figure 2) may affect lipid metabolism. Accordingly, lipid droplet count, as stained by Oil red O color in LPS-tolerant FcgRIIB–/– macrophage at 24 h after stimulation, was higher than other groups (Figures 3D,E). This is in contrast to mitochondrial biogenesis, ATP production (Figures 2B–D), and extra cellular flux analysis (Figures 3A,B). Of note, lipid droplets were not increased in single LPS-stimulated FcgRIIB–/– macrophages (Figures 3D,E). Furthermore, untargeted LC-MS-based lipidomic analysis was performed. The schematic diagram of the analysis is detailed in Figure 4A. Increased expression of glycerolipid (GL), glycerophospholipid (GP), sphingosine (SP), and sterol (ST) in LPS-tolerant FcgRIIB–/– macrophages (100/100) compared with LPS-tolerant WT cells was visualized by clustering heat map analysis (Figure 4B, right side). In parallel, reduced GP and increased SP and ST in single LPS stimulated FcgRIIB–/– cells (N/100) were observed when compared with WT (Figure 4B, left side). A Venn diagram of lipid derivatives from LPS-tolerant FcgRIIB–/– macrophages (100/100) vs. single LPS stimulation (N/100) revealed 47 and 49 lipids, respectively. There were only eight lipids that shared similarity between groups (Figure 4C). This implies different lipid metabolism between these groups. A list of lipid derivatives (lipids) in single and sequential LPS-stimulated macrophages identified by the Lipidomic Gateway database are shown in Tables 2, 3. Among the significantly different lipid derivatives between groups, 20 and 29 lipids were up- and downregulated, respectively, in FcgRIIB–/– macrophages with single LPS stimulation (Table 2). While 39 and 11 lipids in FcgRIIB–/– macrophages with LPS-tolerance were up- and down- regulated, respectively (Table 3). These alterations mostly occur in glycerophospholipids. Raw data of lipidomic analysis in LPS-tolerant macrophages is included in Supplemental Data.

**Table 2:** Summary of lipid derivatives based on lipidomic gateway analysis with mass spectrometric measurement identification: comparison of lipid profiles of FcgRIIB–/– and FcgRIIB+/+ macrophages with single LPS stimulation (N/100).

				**Intensity (m/z)**			
**Compound**	**Formula**	**Ion**	**Category**	**FcgRIIB–/–**	**FcgRIIB+/+**	*p-*value^*^	**Regulation#**
Halaminol A	C14H30NO	[M+H]+	Sterolipids	155,650 ± 1,258	218,881 ± 2,045	0.00006	Up
C19 Sphingosine-1-phosphate	C19H39NO4P	[M+H-H2O]+	Sphingolipids	5,413 ± 231	10,700 ± 108	0.00035	Up
PI(O-16:0/14:0)	C39H76O11P	[M+H-H2O]+	Glycerophospholipids	1,362 ± 56	2,210 ± 54	0.0004	Up
PI(P-16:0/0:0)	C25H49O11PNa	[M+Na]+	Glycerophospholipids	8,278 ± 242	14,536 ± 398	0.00054	Up
PE(22:0/0:0)	C27H57NO7P	[M+H]+	Glycerophospholipids	881 ± 41	1,417 ± 40	0.00071	Up
PE(19:0/0:0)	C24H50NO7P	[M+H]+	Glycerophospholipids	16,526 ± 127	29,995 ± 289	0.00135	Up
PC(O-16:0/0:0)	C24H53NO6P	[M+H]+	Glycerophospholipids	1,034,750 ± 9,895	1,293,654 ± 22,371	0.00261	Up
PG(18:4(6Z,9Z,12Z,15Z)/13:0)	C37H64O9P	[M+H-H2O]+	Glycerophospholipids	1,211 ± 68	3,239 ± 256	0.00613	Up
PC(0:0/18:1(9E))	C26H52NO7P	[M+H]+	Glycerophospholipids	6,502 ± 152	9,787 ± 380	0.00649	Up
PC(18:0/0:0)	C26H55NO7P	[M+H]+	Glycerophospholipids	1,099 ± 47	1,721 ± 90	0.00869	Up
PE(20:0/0:0)	C25H53NO7P	[M+H]+	Glycerophospholipids	20,662 ± 1,014	29,342 ± 1,380	0.00893	Up
PC(P-18:0/0:0)	C26H55NO6P	[M+H]+	Glycerophospholipids	4,206 ± 128	6,898 ± 218	0.00917	Up
PC(0:0/18:1(6Z))	C26H52NO7P	[M+Na]+	Glycerophospholipids	2,172 ± 235	4,161 ± 691	0.00965	Up
PE(18:0/0:0)	C23H48NO7PNa	[M+Na]+	Glycerophospholipids	62,669 ± 263	114,196 ± 2,124	0.00995	Up
1-O-(2-methoxy-hexadecyl)-sn-glycerol	C20H42O4Na	[M+Na]+	Glycerolipids	9,065 ± 150	13,224 ± 519	0.01045	Up
PC(P-16:0/17:1(9Z))	C41H80NO7PK	[M+K]+	Glycerophospholipids	69,884 ± 1,489	78,782 ± 1,328	0.01149	Up
PC(16:0/0:0)	C24H50NO7PNa	[M+Na]+	Glycerophospholipids	1,609 ± 91	2,857 ± 199	0.013	Up
PC(P-16:0/0:0)	C24H51NO6P	[M+H]+	Glycerophospholipids	998 ± 9	1,472 ± 94	0.03591	Up
Taccalonolide A	C36H45O13	[M+H-H2O]+	Sterolipids	13,424 ± 237	15,225 ± 443	0.03597	Up
PC(18:1(9E)/2:0)	C28H54NO8P	[M+H-H2O]+	Glycerophospholipids	962 ± 76	1,572 ± 155	0.04055	Up
PA(16:0/14:0)	C33H65O8PK	[M+K]+	Glycerophospholipids	12,147 ± 125	8,148 ± 191	0.00017	Down
2-linoleoyl-sn-glycerol	C21H38O4	[M+NH4]+	Glycerolipids	2,689 ± 52	999 ± 21	0.00021	Down
PE(16:0/0:0)	C21H44NO7PNa	[M+Na]+	Glycerophospholipids	5,832 ± 163	3,124 ± 141	0.00026	Down
16,17-didehydropregnenolone	C21H30O2K	[M+K]+	Sterolipids	9,000 ± 168	5,804 ± 193	0.00026	Down
3a,17a-Dihydroxy-5b-androstane	C19H32O2K	[M+K]+	Sterolipids	11,000 ± 261	4,087 ± 114	0.00028	Down
PE(0:0/20:5(5Z,8Z,11Z,14Z,17Z))	C25H42NO7P	[M+H]+	Glycerophospholipids	14,400 ± 45	6,040 ± 192	0.00028	Down
PE(24:6(6Z,9Z,12Z,15Z,18Z, 21Z)/0:0)	C29H49NO7P	[M+H]+	Glycerophospholipids	1,566 ± 58	604 ± 39	0.00035	Down
MG(18:2(9Z,12Z)/0:0/0:0)[rac]	C21H38O4	[M+H-H2O]+	Glycerolipids	9,473 ± 269	5,786 ± 226	0.00054	Down
1-O-(2R-hydroxy-pentadecyl)-sn-glycerol	C18H38O4Na	[M+Na]+	Glycerolipids	3,051 92	1,802 ± 78	0.00056	Down
Dinorlithocholic acid	C22H40NO3	[M+NH4]+	Sterolipids	66,119 ± 11,380	19,952 ± 1,954	0.0006	Down
PE(22:4(7Z,10Z,13Z,16Z)/0:0)	C27H49NO7P	[M+H]+	Glycerophospholipids	1,737 ± 65	928 ± 47	0.00084	Down
PE(20:5(5Z,8Z,11Z,14Z,17Z)/0:0)	C25H42NO7PNa	[M+Na]+	Glycerophospholipids	1,819 ± 35	779 ± 12	0.00113	Down
1alpha,25-dihydroxy-21-nor-20-oxavitam	C25H44NO4	[M+NH4]+	Sterolipids	1,425 ± 344	789 ± 44	0.00149	Down
Cer(d18:1/2:0)	C20H39NO3Na	[M+Na]+	Sphingolipids	12,387 ± 456	5,162 ± 129	0.00224	Down
PE(18:2(9Z,12Z)/0:0)	C23H44NO7P	[M+H]+	Glycerophospholipids	9,532 ± 321	3,359 ± 43	0.00234	Down
3-ketosphinganine	C18H36NO	[M+H-H2O]+	Sphingolipids	64,307 ± 423	41,913 ± 1,468	0.0024	Down
1alpha,25-dihydroxy-19-nor-22-oxavitam	C25H46NO4	[M+NH4]+	Sterolipids	3,109 ± 55	2,244 ± 53	0.0032	Down
12-Oxo-5alpha-cholan-24-oic Acid	C24H38O3	[M+NH4]+	Sterolipids	10,217 ± 451	5,319 ± 171	0.00388	Down
PE(20:4(5Z,8Z,11Z,14Z)/0:0)	C25H45NO7P	[M+H]+	Glycerophospholipids	5,535 ± 148	3,689 ± 40	0.00405	Down
PE(22:5(4Z,7Z,10Z,13Z,16Z)/0:0)	C27H46NO7PNa	[M+Na]+	Sphingolipids	32,509 ± 259	25,847 ± 889	0.01209	Down
POV-PA	C24H49NO9P	[M+NH4]+	Glycerophospholipids	28,518 ± 833	23,737 ± 543	0.01242	Down
MG(0:0/20:4(5Z,8Z,11Z,14Z)/0:0)	C23H37O3	[M+H-H2O]+	Glycerolipids	6,099 ± 230	4,592 ± 96	0.01248	Down
PS(16:1(9Z)/22:2(13Z,16Z))	C44H80NO10PN	[M+Na]+	Glycerophospholipids	11,108 ± 888	4,814 ± 173	0.01648	Down
PC(16:0/2:0)	C26H51NO7P	[M+H-H2O]+	Glycerophospholipids	1,320 ± 72	798 ± 107	0.02021	Down
Sphingosine	C18H38NO2	[M+H]+	Sphingolipids	1,177 ± 42	962 ± 32	0.02255	Down
PC(20:2(11Z,14Z)/0:0)	C28H54NO7PNa	[M+Na]+	Glycerophospholipids	3,152 ± 164	2,146 ± 12	0.02515	Down
Sphinganine	C18H38NO	[M+H-H2O]+	Sphingolipids	1,581 ± 148	730 ± 43	0.02521	Down
1-(2-methoxy-6Z-heptadecenyl)-sn-glyce	C23H49NO7P	[M+H]+	Glycerophospholipids	1,014 ± 35	858 ± 21	0.0274	Down
Sphingosine-1-phosphate	C18H37NO4P	[M+H-H2O]+	Sphingolipids	1,234 ± 46	964 ± 39	0.0308	Down

* *p < 0.05 FcgRIIB–/– vs. FcgRIIB+/+; #, direction of change in FcgRIIB–/– vs. FcgRIIB+/+*.

**Table 3:** Summary of lipid derivatives based on lipidomic gateway analysis with mass spectrometric measurement identification: comparison of lipid profile of FcgRIIB–/– and FcgRIIB+/+ macrophages with sequential LPS stimulation (100/100).

				**Intensity (m/z)**	
**Compound**	**Formula**	**Ion**	**Category**	**FcgRIIB–/–**	**FcgRIIB+/+**	*p-*value^*^	**Regulation#**
PI(18:1(9Z)/0:0)	C27H51O12P	[M+H]+	Glycerophospholipids	10,111 ± 418	4,037 ± 356	0.00044	Up
PE(18:0/0:0)	C23H47NO6P	[M+H-H2O]+	Glycerophospholipids	11,731 ± 528	4,683 ± 327	0.00088	Up
PA(12:0/14:0)	C29H61NO8P	[M+NH4]+	Glycerophospholipids	4,970 ± 206	1,769 ± 266	0.0009	Up
POV-PA	C24H49NO9P	[M+NH4]+	Glycerophospholipids	67,817 ± 2,890	18,364 ± 1,193	0.00104	Up
PE(18:1(11Z)/0:0)	C23H47NO7P	[M+H]+	Glycerophospholipids	10,818 ± 567	4,489 ± 396	0.00132	Up
PC(0:0/18:1(9Z))	C26H53NO7P	[M+H]+	Glycerophospholipids	12,620 ± 574	6,270 ± 565	0.0014	Up
CPA(18:0)	C21H45NO6P	[M+NH4]+	Glycerophospholipids	42,165 ± 1,515	24,175 ± 1,674	0.0014	Up
PE(16:0/0:0)	C21H45NO7P	[M+H]+	Glycerophospholipids	27,108 ± 3,887	6,325 ± 454	0.00164	Up
PI(20:4(5Z,8Z,11Z,14Z)/0:0)	C29H49O12PNa	[M+Na]+	Glycerophospholipids	3,039 ± 158	1,690 ± 124	0.0031	Up
1-(2-methoxy-6Z-heptadecenyl)-sn-g	C23H49NO7P	[M+H]+	Glycerophospholipids	165,910 ± 8,158	65,591 ± 2,816	0.00323	Up
1-(2-methoxy-6Z-octadecenyl)-sn-gly	C24H51NO7P	[M+H]+	Glycerophospholipids	31,731 ± 1,377	18,167 ± 1,601	0.00326	Up
PE(18:2(9Z,12Z)/0:0)	C23H45NO7P	[M+H]+	Glycerophospholipids	6,852 ± 419	2,183 ± 165	0.00345	Up
PE(0:0/20:3(11Z,14Z,17Z))	C25H47NO7P	[M+H]+	Glycerophospholipids	9,856 ± 490	4,819 ± 221	0.00346	Up
MG(18:2(9Z,12Z)/0:0/0:0)[rac]	C21H42NO4	[M+NH4]+	Glycerolipids	3,843 ± 168	2,371 ± 94	0.00395	Up
C17 Sphinganine	C17H38NO2	[M+H]+	Sphingolipids	48,051 ± 3,025	6,969 ± 444	0.00465	Up
PC(16:1(9Z)/0:0)	C24H49NO7P	[M+H]+	Glycerophospholipids	3,700 ± 198	2,262 ± 171	0.00572	Up
PC(20:1(11Z)/0:0)	C28H57NO7P	[M+H]+	Glycerophospholipids	3,947 ± 224	2,396 ± 171	0.00646	Up
1-(2-methoxy-5Z-hexadecenyl)-sn-gly	C22H47NO7P	[M+H]+	Glycerophospholipids	5,112 ± 303	2,600 ± 130	0.00668	Up
PG(P-18:0/0:0)	C24H53NO8P	[M+NH4]+	Glycerophospholipids	5,074 ± 383	2,186 ± 95	0.00849	Up
PI(P-18:0/0:0)	C27H54O11P	[M+H]+	Glycerophospholipids	3,420 ± 228	1,394 ± 49	0.00984	Up
PG-PG	C27H51O12PNa	[M+Na]+	Glycerophospholipids	96,567 ± 662	5,593 ± 656	0.0101	Up
POV-PG	C27H52O11P	[M+H]+	Glycerophospholipids	25,827 ± 2,012	12,884 ± 1,066	0.01041	Up
1-(2-methoxy-13-methyl-6Z-tetradec	C22H45NO9P	[M+H]+	Glycerophospholipids	8,387 ± 771	2,493 ± 180	0.01309	Up
PC(P-18:0/0:0)	C26H55NO6P	[M+H]+	Glycerophospholipids	88,434 ± 734	4,488 ± 754	0.01441	Up
PI(O-18:0/0:0)	C27H56O11P	[M+H]+	Glycerophospholipids	2,241 ± 195	1,097 ± 81	0.01669	Up
PE(P-18:0/0:0)	C23H49NO6P	[M+H]+	Glycerophospholipids	12,713 ± 1,050	7,280 ± 513	0.02018	Up
PE(0:0/22:5(4Z,7Z,10Z,13Z,16Z))	C27H47NO7P	[M+H]+	Glycerophospholipids	49,295 ± 3,189	31,897 ± 1,223	0.02097	Up
3-ketosphinganine	C18H36NO	[M+H-H2O]+	Sphingolipids	25,083 ± 1,925	13,497 ± 430	0.02217	Up
PI(O-16:0/0:0)	C25H51O11PNa	[M+Na]+	Glycerophospholipids	8,319 ± 886	3,302 ± 164	0.02651	Up
PC(18:1(11Z)/0:0)	C26H52NO7PNa	[M+Na]+	Glycerophospholipids	2,725 ± 147	1,957 ± 173	0.02885	Up
1-(2-methoxy-nonadecanyl)-sn-glyce	C25H53NO6P	[M+H-H2O]+	Glycerophospholipids	1,505 ± 88	1,071 ± 98	0.03033	Up
Sphinganine-1-phosphocholine	C23H55N3O5P	[M+NH4]+	Sphingolipids	1,028 ± 30	899 ± 18	0.03139	Up
PI(18:0/0:0)	C27H54O12P	[M+H]+	Glycerophospholipids	30,517 ± 3,132	15,545 ± 797	0.03438	Up
PE(22:2(13Z,16Z)/0:0)	C27H52NO7PNa	[M+Na]+	Glycerophospholipids	5,334 ± 677	1,894 ± 94	0.03444	Up
Sphingosine	C18H38NO2	[M+H]+	Sphingolipids	24,517 ± 1,557	13,190 ± 247	0.03854	Up
Homochenodeoxycholic acid	C25H42O4	[M+NH4]+	Sterol lipids	2,662 ± 315	1,439 ± 167	0.04069	Up
1-O-(2-methoxy-4Z-hexadecenyl)-sn-	C25H53NO7P	[M+H]+	Glycerophospholipids	3,239 ± 227	2,354 ± 184	0.04107	Up
MG(0:0/20:4(5Z,8Z,11Z,14Z)/0:0)	C23H37O3	[M+H-H2O]+	Glycerolipids	6,632 ± 825	1,951 ± 222	0.04491	Up
DG(16:1(9Z)/22:6(4Z,7Z,10Z,13Z,16Z,1	C41H70NO5	[M+NH4]+	Glycerolipids	3,015 ± 70	5,661 ± 40	0.00004	Down
PE(12:0/0:0)	C42H67O8PNa	[M+Na]+	Glycerophospholipids	1,268 ± 36	2,191 ± 61	0.00067	Down
MG(0:0/20:5(5Z,8Z,11Z,14Z,17Z)/0:0)	C43H71O5	[M+H]+	Glycerolipids	7,846 ± 441	14,896 ± 694	0.00205	Down
16,17-didehydroprogesterone	C25H44NO7P	[M+H]+	Glycerophospholipids	13,306 ± 472	22,673 ± 944	0.00327	Down
ST 25:4;O5;T	C18H28O2K	[M+K]+	Sterol lipids	30,089 ± 1,980	71,772 ± 4,231	0.00369	Down
Estrone	C27H41NO7S	[M+H]+	Sterol lipids	33,834 ± 2.081	53,084 ± 3,034	0.00886	Down
PE(20:4(5Z,8Z,11Z,14Z)/0:0)	C23H36O4Na	[M+Na]+	Glycerolipids	2,034 ± 132	2,709 ± 110	0.0183	Down
DG(18:1(9Z)/22:6(4Z,7Z,10Z,13Z,16Z,1	C17H37NO7P	[M+H]+	Glycerophospholipids	1,041 ± 37	1,293 ± 53	0.0219	Down
PA(17:2(9Z,12Z)/22:6(4Z,7Z,10Z,13Z,1	C21H32NO2	[M+NH4]+	Sterol lipids	4,140 ± 380	6,764 ± 595	0.02732	Down
19-norandrosterone	C27H49NO7P	[M+H]+	Glycerophospholipids	13,207 ± 302	15,902 ± 649	0.03638	Down
PE(22:4(7Z,10Z,13Z,16Z)/0:0)	C18H22O2K	[M+K]+	Sterol lipids	5,097 ± 185	10,229 ± 1,200	0.04762	Down

* *p < 0.05 FcgRIIB–/– vs. FcgRIIB+/+; #, direction of change in FcgRIIB–/– vs. FcgRIIB+/+*.

**Figure 5:**
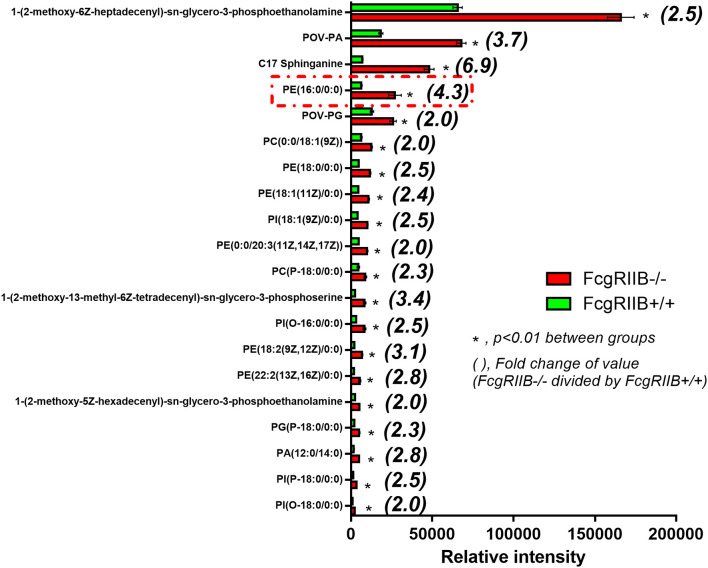
Top first 20 high intensity lipid derivatives in macrophages after LPS-tolerance (100/100) from FcgRIIB–/– vs. wild-type (FcgRIIB+/+) identified by lipids map gateway with a cut off *p*-value < 0.01 together with the difference of values with more than 1.5 fold-change is demonstrated (Independent triplicate experiments were performed to prepare cells for lipidomic analysis).

Lipids in LPS-tolerant macrophages in FcgRIIB–/– and WT were further analyzed as a result of mitochondrial defect where energy depletion and increased cellular lipid content (Figures 2, 3) were more dominant in LPS-tolerant macrophages compared with single LPS-stimulated cells. Firstly, principle component analysis (PCA) of lipid derivatives showed a clear separation between LPS-tolerant FcgRIIB–/– macrophages (100/100) compared with WT. PCA analysis also identified a 39% principle component 1 (PC1) measurement of variance, which suggests the variability of lipids in WT and FcgRIIB–/– macrophages (Figure 4D). Secondly, a Volcano plot demonstrated the distribution of up- and downregulated lipids with a cut off *p*-value at 0.05 and log_2_FC at 1.5 folds difference between LPS-tolerant FcgRIIB–/– macrophages vs. WT cells (Figure 4E). Thirdly, among the upregulated lipid derivatives of FcgRIIB–/– vs. WT cells, biological significance from pathway enrichment analysis identified that phospholipid biosynthesis was the most significantly enriched lipid (Figure 4F, red), followed by the derivatives in arachidonic acid metabolism (Figure 4F, yellow). Fourthly, the lists of upregulated lipids were further explored and analyzed by map-gateway analysis. The top 20 abundant lipids among 39 upregulated lipid derivatives of LPS-tolerant FcgRIIB–/– macrophages over WT (Table 3 and Supplemental Data) with *p* value differences of less than 0.01, and ratios (FcgRIIB–/– divided by FcgRIIB+/+) of more than 1.5 fold in FcgRIIB–/– macrophages over WT are demonstrated in Figure 5. Most of these lipids were in glycerophospholipid pathways. Although the abundance of 2-methoxy-6Z-heptadecenyl-sn-glycero-3-PE of LPS-tolerant FcgRIIB–/– macrophages was the highest (top column of Figure 5), the increase was only 2.5 folds higher than WT. In addition, the second and third most abundant derivatives were C17 Sphinganine at 6.9 folds and POV-PA at 3.7 folds higher than WT, respectively. However, C17 Sphinganine, as a sphingolipid derivative, and POV-PA, as an oxidized phospholipid, were not enriched in phospholipid biosynthesis or the arachidonic acid metabolism pathway that was derived from the pathway analysis (Figure 4F). This implies biologically less meaningful patterns. PE (16:0/0:0) (PE) was the fourth most abundant lipid at 4.3 folds higher in FcgRIIB–/– macrophage compared with WT and became the focus for further study (Figure 5, red dotted line). Indeed, phosphatidylcholines (PC) and phosphatidylyethanolamine (PE) are major lipid derivatives in the GP group (37, 38). PE is also considered as one of the major phospholipid components of eukaryotic cell membrane (39). Enriched PE component in LPS-tolerant FcgRIIB–/– macrophages over WT was possibly due to increased lipid uptake or enhanced lipid synthesis.

### Involvement of Phosphatidylethinolamine Methyltransferease (PEMT) and AMPK in LPS-Tolerant FcgRIIB–/– Macrophages

**Figure 6:**
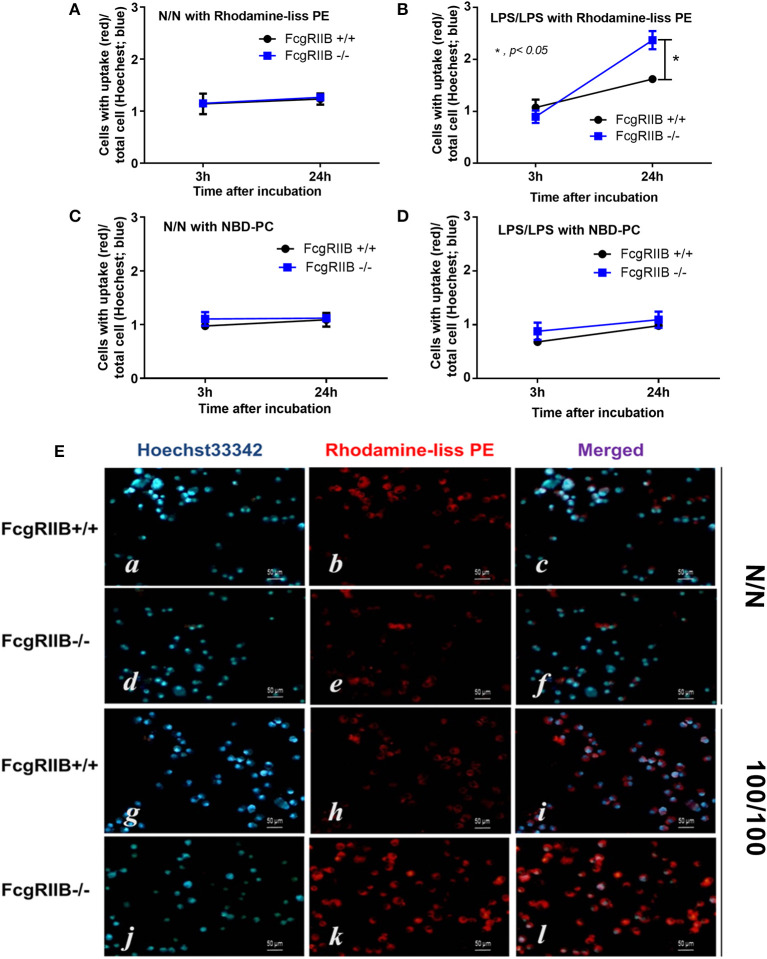
Uptake of lipid analogs Rhodamine-liss phosphatidylethanolamine (Rhodamine-liss PE) **(A,B)** or fluorescent phosphatidylcholine (NBD-PC) **(C,D)** normalized by Hoechst 33342 nucleus staining, in FcgRIIB–/– and wild-type (FcgRIIB+/+) macrophages in control (N/N) and LPS-tolerance (100/100) with representative immunofluorescence images **(E)** are demonstrated (images of NBD-PC uptake are not shown). (Independent triplicate experiments were performed).

Technical limitations in the evaluation of intracellular lipid synthesis (29, 30) resulted in performing only the lipid uptake assay. Accordingly, uptake of PE analog (Rhodamine-liss PE) was higher in LPS-tolerant FcgRIIB–/– macrophages when compared with WT at 24h of incubation, while the uptake of phosphatidylcholine (PC) analog (NBD-PC) was similar between WT and FcgRIIB–/– macrophages (Figure 6). Although mechanisms of enhanced-uptake of Rhodamine-liss PE in LPS-tolerant FcgRIIB–/– macrophages were not clear, these results suggested an association between PE and LPS-tolerance in macrophages of lupus mice.

**Figure 7:**
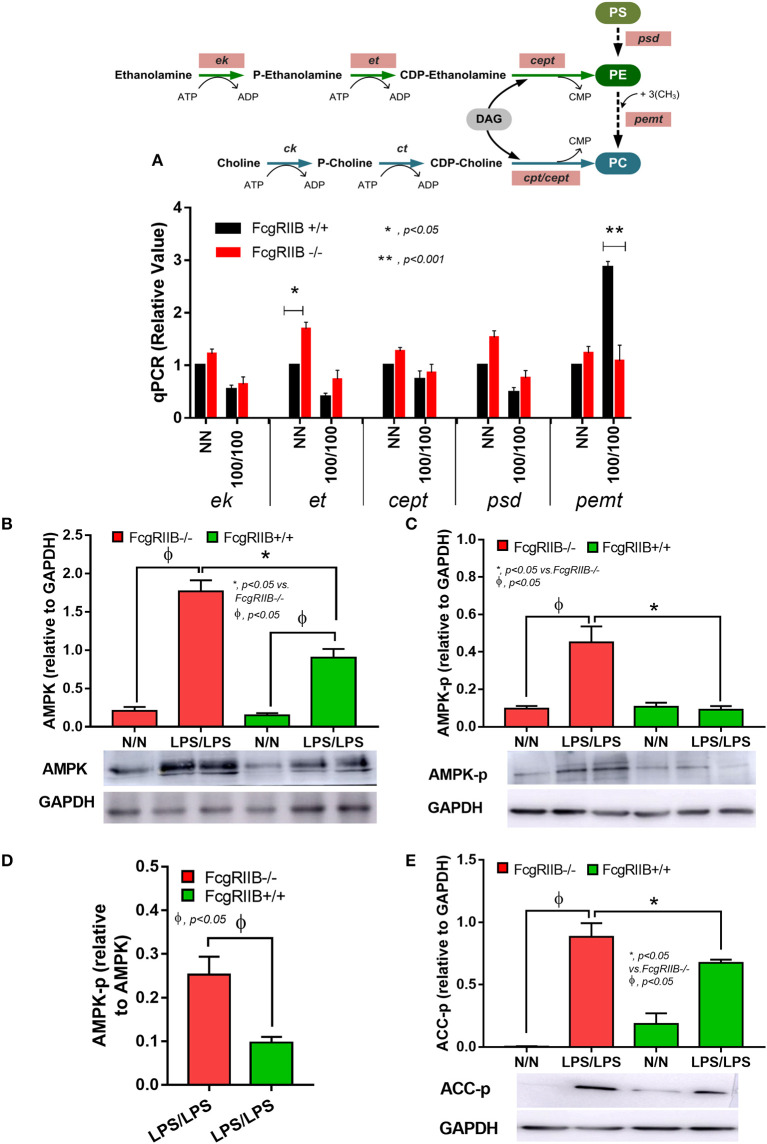
Schematic diagram of important enzymes including ethanolamine kinase (*ek*), CTP: phosphoethanolamine cytidylyltransferase (*et*), choline/ethanolamine phosphotransferase (*cept*), phosphatidylserine decarboxylase (*psd*), phosphatidylethanolamine N-methyltransferase (*pemt*), and CTP-phosphocholine cytidylyltransferase/choline/ethanolamine phosphotransferase (*cpt/cept*) in lipogenesis pathway with phosphatidylserine (PS), phosphatidylyethanolamine (PE), and phosphatidylcholine (PC) (**A**, upper part) and gene expression from macrophages from FcgRIIB–/– and wild-type (FcgRIIB+/+) in control (N/N) or LPS-tolerance (100/100) (**A**, lower part) are demonstrated (DAG, diacyl glycerol). Protein abundance of macrophages in control (N/N) or LPS-tolerance (100/100) as determined by AMP-activated protein kinase (AMPK) in relative to Glyceraldehyde 3-phosphate dehydrogenase (GAPDH) **(B)**, phosphorylated AMPK (AMPK-p) in relative to GAPDH **(C)**, AMPK-p abundance in relative to AMPK **(D)**, and phosphorylated acetyl-CoA carboxylase (ACC-p) **(E)** of LPS-tolerant macrophages with representative pictures of Western blot analysis are also demonstrated (Independent triplicate experiments were performed).

Since several enzymes are needed for PE and PC lipogenesis (Figure 7, diagram), the expression of these enzymes between LPS-tolerant FcgRIIB–/– macrophages vs. WT cells were examined. Interestingly, there was high expression of phosphoethanolamine cytidylyltransferase (*et*), the encoding enzyme responsible for PE synthesis, at the baseline condition (N/N) of FcgRIIB–/– macrophages compared with WT (Figure 7A). LPS-tolerant FcgRIIB–/– macrophages demonstrated a decreased expression of phosphatidylethanolamine N-Methyltransferase (*pemt*) (Figure 7A), which is the encoding enzyme responsible for the conversion of PE into PC. Indeed, the reduced *pemt* expression (Figure 7A), and increased PE uptake (Figure 6) in LPS-tolerant FcgRIIB–/– macrophages is consistent with the higher intracellular PE (Figure 5) in LPS-tolerant FcgRIIB–/– macrophages compared with WT.

**Figure 8:**
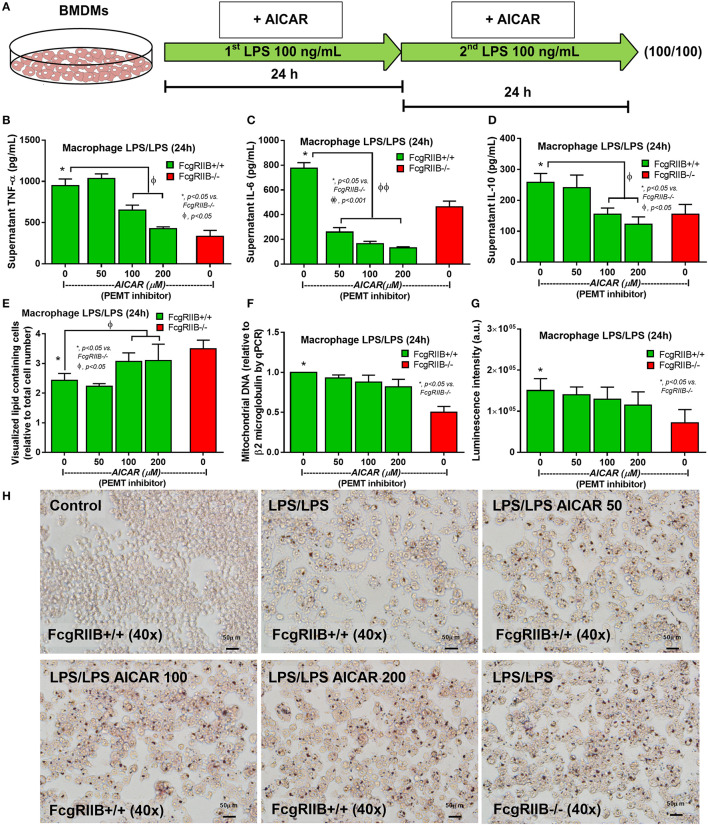
Schematic diagram **(A)** of the experiment with LPS-tolerance induction (LPS/LPS) in wild-type (FcgRIIB+/+) bone marrow-derived macrophages (BMDMs) with aminoimidazole-4-carboxamide ribonucleotide (AICAR; a *pemt* inhibitor) compared with LPS-tolerant FcgRIIB–/– macrophages as determined by supernatant cytokines **(B–D)**, number of lipid droplets intensity from Oil Red O staining by ImageJ software evaluation **(E)**, semi-quantitative expression of mitochondrial DNA content (mtDNA) relative to β2 microglobulin (β2M) gene by qRT-PCR **(F)**, luminescence intensity of cellular ATP production **(G)**, and the representative Oil Red O staining **(H)** from these cells after the stimulations are demonstrated. Independent triplicate experiments were performed.

Activation status of AMPK in LPS-tolerance was also explored because of the well-known association between PEMT pathway of lipid metabolism and the sensor of cellular energy status, AMP-activated protein kinase (AMPK) (40, 41). Accordingly, higher protein burdens of AMPK, phosphorylated AMPK (AMPK-p), and phosphorylated acetyl-CoA carboxylase (ACC-p, a downstream signaling of AMPK) in LPS-tolerant FcgRIIB–/– macrophages compared with WT cells was observed (Figures 7B–E). Of note, burdens of AMPK but not AMPK-p, was also increased in LPS-tolerant WT macrophages (Figure 7B). Hence, prominent *pemt* reduction and increased AMPK-p in response to energy depletion of LPS-tolerant FcgRIIB–/– macrophages compared with WT might be responsible for the severe unresponsiveness against the second dose of LPS in LPS-tolerant FcgRIIB–/– cells. To address the role of PEMT, 5-aminoimidazole-4-carboxamide-1-β-D-ribofuranoside (AICAR), an inhibitor of *pemt* (in low dose; <500 μM) with the AMPK enhancer property (in high dose) (42), was incubated in LPS-tolerant WT macrophages to see if AICAR could enhance severity of LPS exhaustion as seen in LPS-tolerant FcgRIIB–/– macrophages. As expected, AICAR worsened LPS-tolerance in WT macrophages in a dose-dependent manner as demonstrated by cytokine reduction with increased cellular lipid droplets, similar to LPS-tolerant FcgRIIB–/– cells (Figures 8A–E,H). However, AICAR did not alter cell energy as determined by mtDNA and ATP production (Figures 8F,G). This is possibly due to the selected doses were not high enough to induce AMPK.

**Figure 9:**
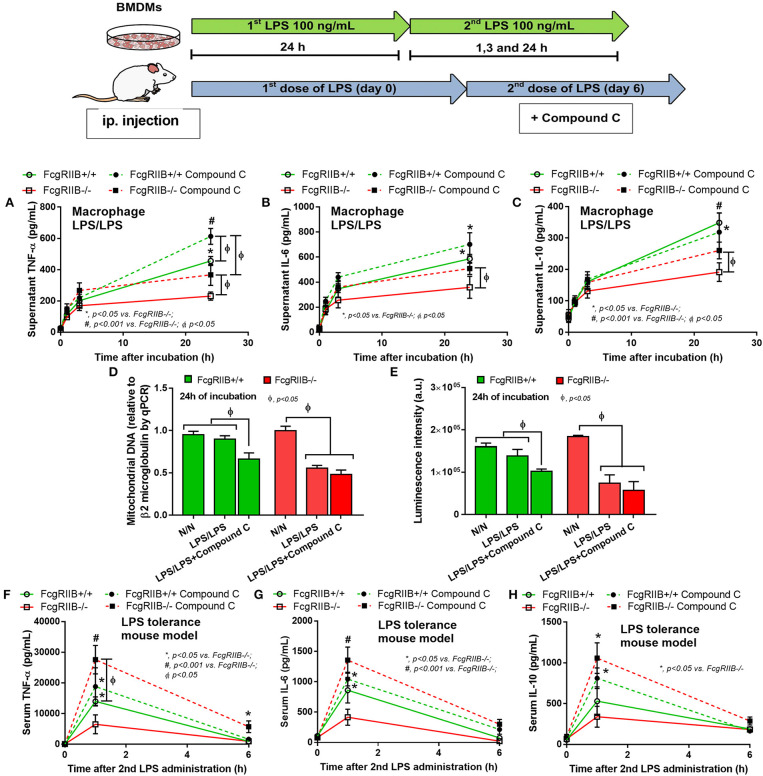
Schematic diagram of the experiment with LPS-tolerance induction (LPS/LPS) in macrophages and LPS injection in mice are shown (upper part). Supernatant cytokines of LPS-tolerant FcgRIIB–/– macrophages and wild-type (FcgRIIB+/+) with or without compound C (AMPK inhibitor) **(A–C)**, semi-quantitative expression of mitochondrial DNA content (mtDNA) relative to β2 microglobulin (β2M) gene by qRT-PCR **(D)**, and luminescence intensity of cellular ATP production **(E)** are demonstrated (independent triplicate experiments were performed). Serum cytokine from FcgRIIB–/– and wild-type mice after LPS-tolerance induction with placebo control or compound C treatment **(F–H)** are shown (*n* = 6–7/group). BMDMs, bone marrow-derived macrophages.

The known association between PEMT and AMPK (40, 41) together with prominent AMPK-p in LPS-tolerant FcgRIIB–/– macrophages of lupus mice (Figure 7C) makes AMPK an interesting target to harness LPS-tolerance in lupus. To test the role of AMPK in LPS-tolerance, more experiments were performed using Compound C, a specific AMPK inhibitor. As such, Compound C was given during LPS challenge and increased supernatant TNF-α in LPS-tolerant WT macrophages. It enhanced all cytokines in LPS-tolerant FcgRIIB–/– macrophages (Figures 9A–C). Compound C reduced mtDNA and cellular ATP in LPS-tolerant WT macrophages but not in FcgRIIB–/– cells (Figures 9D,E). In addition, Compound C also attenuated LPS-tolerance in FcgRIIB–/– mice as determined by serum cytokines at 1 h after the second LPS injection. However, it was not effective in LPS-tolerance on WT mice (Figures 9F–H).

## Discussion

FcgRIIB–/– mice provide a good representative lupus model for Asian population due to the high prevalence of FcgRIIB dysfunction-polymorphisms (43). Persistent LPS exposure due to active lupus induced spontaneous endotoxemia (13, 14) possibly induces extreme LPS exhaustion with increased susceptibility to secondary infection (6). Here, we demonstrated that prominent LPS-tolerance in FcgRIIB–/– macrophages is, at least in part, due to an alteration in lipid-derivative metabolism. In a translational implication, an AMPK inhibitor rescued LPS-tolerance in lupus mice and might be a candidate for treatment of frequent infections in patients with lupus.

### Severe LPS-Tolerance, Mitochondrial Defects, and Lipid Accumulation in FcgRIIB–/– Macrophages

FcgRIIB–/– macrophages showed hyper-responsiveness after a single LPS stimulation but more severe depressed cytokine production after subsequent doses of LPS (referred to as “LPS-tolerance”) in comparison with WT cells (6, 7). Immense exhaustion of cytokine production in LPS-tolerant FcgRIIB–/– macrophages compared with WT was possibly associated with decreased cell energy as determined by mitochondrial biogenesis, mtDNA, ATP production, and extracellular flux analysis. In contrast, energy status of single LPS-stimulated FcgRIIB–/– macrophages was not higher than LPS-stimulated WT cells. This indicates that LPS hyper-responsiveness in FcgRIIB–/– macrophages is more complicated than energy status alone. It could be that cell energy of hyper-responsive FcgRIIB–/– macrophages increases in a short period of time before robustly exhausting. This can be visualized by repetitive LPS-stimulations. Our data suggests that prominent energy exhaustion after the second dose of LPS in FcgRIIB–/– macrophages might be responsible from the hyper-responsive responses against first LPS challenge. Accordingly, defect in mitochondrial Krebs cycle (36 ATP production) and glycolysis (2 ATP production) in leukocytes with LPS-tolerance is reported (18, 19).

Lipid β-oxidation is a part of the cell energy process (22) where energy depletion in LPS-tolerant macrophages might be associated with lipid metabolism. Indeed, increased lipid accumulation was demonstrated in LPS-tolerant FcgRIIB–/– macrophages as previously mentioned (44) but not in WT cells. This is possibly due to the energy depletion in WT cells not being severe enough. Most of the lipid derivatives in LPS-tolerant FcgRIIB–/– macrophages were upregulated (Table 3), while approximately half of the derivatives were upregulated in single LPS stimulated FcgRIIB–/– macrophages (Table 2) when compared with WT cells. Among intracellularly accumulated lipids, glycerophospholipid (GP), the lipid component of cell membrane and sphingosine (SP), the lipid of cellular energy (45) were both predominant in LPS-tolerant FcgRIIB–/– macrophages when compared with WT cells by mass-spectrometry analysis (LC-MS). Within several derivatives in GP of LPS-tolerant macrophages, phosphatidylethanolamine (PE) is an important lipid component of cell membrane (37, 38). PE alteration could interfere with membrane fluidity, block LPS trans-membrane signaling and deplete cytokine production (46, 47). Additionally, high PE in LPS-tolerant FcgRIIB–/– macrophages might directly reduce cytokine production because PE is an anti-inflammatory lipid-derivative (48–50).

High PE in LPS-tolerant FcgRIIB–/– macrophages is possibly due to increased PE-uptake as demonstrated by PE intracellular-influx and/ or enhanced PE lipogenesis. This is a possible process from decreased expression of *pemt*, which is the enzyme responsible for converting PE into phosphatidylcholine (PC). Alternatively, prominent PE in LPS-tolerant FcgRIIB–/– cells might be due to an increase in PE uptake. Unfortunately, technical limitations on cellular lipogenesis preclude further investigation in this topic (29, 30). On the other hand, low PC in LPS-tolerant FcgRIIB–/– macrophages is possibly from the shortage on PC because it is necessary for cytokine secretion (23, 24). In this study, cytokine secretion of FcgRIIB–/– macrophages toward the first dose LPS was very prominent. Alternatively, a reduction of PC in LPS-tolerant FcgRIIB–/– macrophages might be due to increased PC degradation. Although mechanisms for the alteration of lipid derivatives in LPS-tolerant FcgRIIB–/– macrophages is inconclusive, prominent PE and/ or shortage on PC in LPS-tolerant FcgRIIB–/– macrophages are, at least in part, responsible for lower cytokine levels. AICAR, a *pemt* inhibitor, enhanced LPS-tolerant severity in WT macrophages by dampening cytokine levels and enhancing lipid accumulation into similar levels with LPS-tolerant FcgRIIB–/– macrophages. This suppression of macrophage cytokine production by AICAR has also been previously reported (51, 52). However, *pemt* inhibition did not alter cell energy despite inducing some LPS-tolerant characteristics, including reduced cytokine production and enhanced phagocytosis activity, suggesting the diverse mechanisms of LPS-tolerance. Although direct exploration of PE and PC in AICAR-treated LPS-tolerant macrophages was not determined, our data supported that *pemt* inhibition enhanced the severity of LPS-tolerance in WT macrophages.

### Inadequate PEMT and High AMPK, a Proposed Mechanism of LPS-Tolerance in FcgRIIB–/– Macrophages

While PEMT is encoded by *pemt* and associated with lipogenesis, *pemt* also co-operates with AMPK, a sensor of cellular energy status (40, 41), because AMPK is upregulated in *pemt-*deficient mice (41). Accidentally, AICAR is not only a potent *pemt* inhibitor, but it has also been reported to act as an AMPK activator in high doses (42). Therefore, the cross talk between PEMT and AMPK after LPS activation is possible. As such, LPS induces cytokine production through PEMT*-*mediated lipogenesis (23, 53, 54). From our data, the inhibition of PEMPT by a low dose AICAR reduced cytokine production (42). In addition, LPS promotes fatty acid oxidation by AMPK-dependent-TLR4 activation (23, 53, 54). On the other hand, AMPK activation that occurs during cell stress, including starvation, induces fatty acid translocation into mitochondria for enhancing energy production (55) and directly inhibits cytokine production to possibly restore cell energy (56). Here, we demonstrated increased AMPK in LPS-tolerant macrophages, which was partly responsible for low cytokine production, especially in LPS-tolerant FcgRIIB–/– macrophages. Compound C, an AMPK inhibitor, reduced ATP only in LPS-tolerant WT macrophages but not in FcgRIIB–/– macrophages. This is perhaps due to already minimized cell energy in LPS-tolerant FcgRIIB–/– macrophages where further energy-depletion is prohibited to maintain normal cell-homeostasis (57). Nevertheless, AMPK inhibitor could attenuate LPS-tolerance in macrophages and mice in FcgRIIB–/– groups. This might be an interesting target for harnessing LPS-tolerance in lupus in the future. Indeed, the enhanced cytokine production by Compound C in several situations has also been mentioned (58, 59). However, Compound C was not effective in rescuing LPS-tolerance in WT macrophages and in WT mice because the energy depletion in LPS-tolerant WT group was perhaps not severe enough to upregulate AMPK. Of note, the direct effect of Compound C on cell energy and lipid accumulation in macrophages, *in vivo*, was not evaluated because the cell sorting process might affect these parameters. Due to the possibility that extreme LPS-tolerance in lupus might be associated with increased susceptibility to infections (6), AMPK inhibition is an interesting treatment in such condition. Further studies on Compound C and LPS-tolerance in lupus might provide novel therapeutic insight.

**Figure 10:**
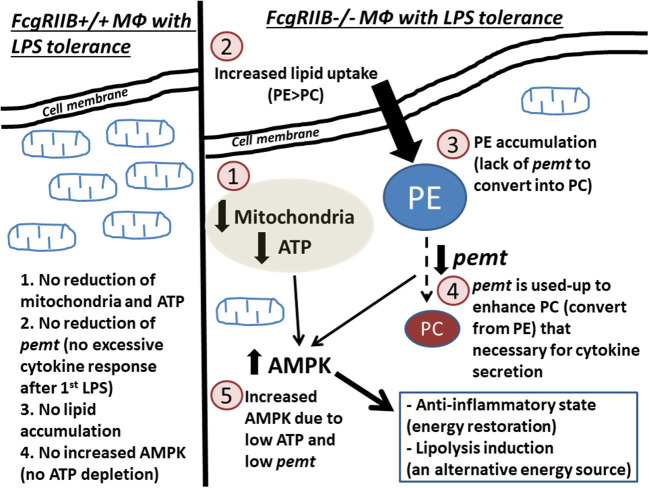
Diagram of the proposed hypothesis is shown. In wild-type macrophages, LPS-tolerance does not induce extreme energy depletion because no exaggerated cytokine production after the first LPS stimulation (left side of the figure). In contrast, in FcgRIIB–/– macrophages (right side of the figure), LPS-tolerance induces severe energy depletion (mitochondria and ATP) (1), which is possibly due to the extreme cytokine production after the first LPS stimulation from inhibitory signaling loss. Subsequently, lipid uptake (2) is increased in response to cell-energy depletion. Conversion from phosphatidylyethanolamine (PE) into phosphatidylcholine (PC) is reduced (3) from inadequate *pemt* (4), which is consumed during profound cytokine secretion process (23, 24) after the first LPS stimulation. In parallel, AMPK (5), in response to ATP depletion, restores cell energy through reduced cytokine production (56) leading to LPS-tolerance.

LPS-tolerance seems to be a characteristic that is inducible by several mechanisms of either energy dependent or energy independent pathways. Our proposed hypothesis on LPS-tolerant FcgRIIB–/– macrophages (Figure 10) initially shows that the first dose of LPS in FcgRIIB–/– macrophages produces higher cytokine production than WT due to inhibitory signaling loss (6, 7). This results in enhancing PEMT utilization for the lipid-associated cytokine secretion process (23). The second dose of LPS had inadequate PEMT to alter PE into PC, which leads to (1) shortage on PC, a lipid-derivative of cytokine secretion process (23, 24), (2) accumulation of PE, an anti-inflammatory lipid-derivative (48–50), and (3) enhancement of AMPK, a signaling that reduces cytokine production to preserve cell energy (53), according to the known association between PEMT and AMPK (41, 60). In parallel, severity of cell energy depletion after the second dose of LPS in FcgRIIB–/– macrophages was enough to induce AMPK. As a metabolic sensor molecule of ATP depletion (53), AMPK sets the anti-inflammatory state to restore cell energy partly by reducing cytokine production (56). Therefore, prominent energy depletion, low PEMT, prominent PE (48–50), reduced PC, and enhanced AMPK (56) are possibly responsible for severe LPS-tolerance in FcgRIIB–/– macrophages. Manipulation of these factors, alone or in combination, might be able to attenuate LPS-tolerance in lupus. As a proof of concept, attenuation of LPS-tolerance in FcgRIIB–/– mice by AMPK inhibitor was demonstrated. Because of the association between LPS-tolerance and infection susceptibility in lupus (6, 7, 21), attenuation of LPS-tolerance might improve the outcome of infectious diseases in patients with lupus.

In conclusion, our results demonstrated an impact of cell-energy depletion and lipid metabolisms in LPS-tolerant FcgRIIB–/– macrophages. We propose that alteration of lipid metabolism is another possible mechanism of LPS-tolerant macrophages, especially in FcgRIIB–/– cells. This finding is in addition to other well-described hypotheses (61). Manipulations in cell energy and/ or lipid metabolisms are potential strategies for harnessing LPS-tolerance in lupus. Further studies are warranted.

## Data Availability Statement

The raw data supporting the conclusions of this article will be made available by the authors, without undue reservation, to any qualified researcher.

## Ethics Statement

The animal study was reviewed and approved by the Institutional Animal Care and Use Committee of the Faculty of Medicine, Chulalongkorn University, Bangkok, Thailand.

## Author Contributions

AL, PR, and TP carried out the conceptualization. TJ, PV, PD, and AL were responsible for the methodology and carried out the investigation. TJ and AL prepared the original draft. AL and TP wrote, reviewed, and edited the manuscript, supervised the study, and acquired the funding.

## Conflict of Interest

The authors declare that the research was conducted in the absence of any commercial or financial relationships that could be construed as a potential conflict of interest.

## References

[1] Bolland S Ravetch JV. Spontaneous autoimmune disease in FcγRIIB-deficient mice results from strain-specific epistasis. Immunity. (2000) 13:277–85. 10.1016/s1074-7613(00)00027-3 10981970

[2] Clatworthy MR Willcocks L Urban B Langhorne J Williams TN Peshu N. Systemic lupus erythematosus-associated defects in the inhibitory receptor FcgammaRIIb reduce susceptibility to malaria. Proc Natl Acad Sci USA. (2007) 104:7169–74. 10.1073/pnas.0608889104 17435165 PMC1855357

[3] Crispin JC Hedrich CM Tsokos GC. Gene-function studies in systemic lupus erythematosus. Nat Rev Rheumatol. (2013) 9:476–84. 10.1038/nrrheum.2013.78 23732569

[4] Clatworthy MR Smith KG. FcgammaRIIb balances efficient pathogen clearance and the cytokine-mediated consequences of sepsis. J Exp Med. (2004) 199:717–23. 10.1084/jem.20032197 14981111 PMC2213308

[5] Maglione PJ Xu J Casadevall A Chan JJ. Fcγ receptors regulate immune activation and susceptibility during *Mycobacterium tuberculosis* infection. J Immunol. (2008) 180:3329–38. 10.4049/jimmunol.180.5.3329 18292558

[6] Ondee T Surawut S Taratummarat S Hirankarn N Palaga T Pisitkun P. Fc gamma receptor IIB deficient mice: a lupus model with increased endotoxin tolerance-related sepsis susceptibility. Shock. (2017) 47:743–52. 10.1097/SHK.0000000000000796 27849678

[7] Ondee T Jaroonwitchawan T Pisitkun T Gillen J Nita-Lazar A Leelahavanichkul A. Decreased protein kinase C-β type II associated with the prominent endotoxin exhaustion in the macrophage of FcGRIIb–/– lupus prone mice is revealed by phosphoproteomic analysis. Int J Mol Sci. (2019) 20:1354. 10.3390/ijms20061354 30889825 PMC6472018

[8] Leentjens J Kox M Koch RM Preijers F Joosten LA van der Hoeven JG. Reversal of immunoparalysis in humans *in vivo*: a double-blind, placebo-controlled, randomized pilot study. Am J Respir Crit Care Med. (2012) 186:838–45. 10.1164/rccm.201204-0645OC 22822024

[9] Hotchkiss RS Monneret G Payen D. Immunosuppression in sepsis: a novel understanding of the disorder and a new therapeutic approach. Lancet Infect Dis. (2013) 13:260–8. 10.1016/S1473-3099(13)70001-X 23427891 PMC3798159

[10] Leelahavanichkul A Somparn P Bootprapan T Tu H Tangtanatakul P Nuengjumnong R. High-dose ascorbate with low-dose amphotericin B attenuates severity of disease in a model of the reappearance of candidemia during sepsis in the mouse. Am J Physiol Regul Integr Comp Physiol. (2015) 309:R223–34. 10.1152/ajpregu.00238.2014 25994956 PMC4525325

[11] Ropes MW. Observations on the natural course of disseminated lupus erythematosus. Medicine. (1964) 43:387–91. 10.1097/00005792-196405000-00016 14168748

[12] Zandman-Goddard G Shoenfeld Y. Infections and SLE. Autoimmunity. (2005) 38:473–85. 10.1080/08916930500285352 16373252

[13] Shi LH Zhang Z Yu AM Wang W Wei Z Akhter E. The SLE transcriptome exhibits evidence of chronic endotoxin exposure and has widespread dysregulation of non-coding and coding RNAs. PLoS ONE. (2014) 9:e93846. 10.1371/journal.pone.0093846 24796678 PMC4010412

[14] Issara-Amphorn J Surawut S Worasilchai N Thim-Uam A Finkelman M Chindamporn A. The synergy of endotoxin and (1–>3)-beta-D-glucan, from gut translocation, worsens sepsis severity in a lupus model of Fc gamma receptor IIb-deficient mice. J Innate Immun. (2018) 10:189–201. 10.1159/000486321 29393221 PMC6757155

[15] Frazier WJ Hall MW. Immunoparalysis and adverse outcomes from critical illness. Pediatr Clin North Am. (2008) 55:647–68. 10.1016/j.pcl.2008.02.009 18501759 PMC2474674

[16] Lopez-Collazo E del Fresno C. Pathophysiology of endotoxin tolerance: mechanisms and clinical consequences. Crit Care. (2013) 17:242. 10.1186/cc13110 24229432 PMC4059412

[17] Hamers L Kox M Pickkers P. Sepsis-induced immunoparalysis: mechanisms, markers, and treatment options. Minerva Anestesiol. (2015) 81:426–39. 24878876

[18] Cheng SC Scicluna BP Arts RJ Gresnigt MS Lachmandas E Giamarellos-Bourboulis EJ. Broad defects in the energy metabolism of leukocytes underlie immunoparalysis in sepsis. Nat Immunol. (2016) 17:406–13. 10.1038/ni.3398 26950237

[19] Arts RJ Gresnigt MS Joosten LA Netea MG. Cellular metabolism of myeloid cells in sepsis. J Leukoc Biol. (2017) 101:151–64. 10.1189/jlb.4MR0216-066R 27272310

[20] Grondman I Arts RJW Koch RM Leijte GP Gerretsen J Bruse N. Frontline science: endotoxin-induced immunotolerance is associated with loss of monocyte metabolic plasticity and reduction of oxidative burst. J Leukocyte Biol. (2019) 106:11–25. 10.1002/Jlb.5hi0119-018r 31169935 PMC6852552

[21] Ondee T Gillen J Visitchanakun P Somparn P Issara-Amphorn J Dang Phi C. Lipocalin-2 (Lcn-2) attenuates polymicrobial sepsis with LPS preconditioning (LPS-tolerance) in FcGRIIb deficient lupus mice. Cells. (2019) 8:1064. 10.3390/cells8091064 31514375 PMC6769833

[22] Houten SM Wanders RJ. A general introduction to the biochemistry of mitochondrial fatty acid beta-oxidation. J Inherit Metab Dis. (2010) 33:469–77. 10.1007/s10545-010-9061-2 20195903 PMC2950079

[23] Tian Y Pate C Andreolotti A Wang L Tuomanen E Boyd K. Cytokine secretion requires phosphatidylcholine synthesis. J Cell Biol. (2008) 181:945–57. 10.1083/jcb.200706152 18559668 PMC2426940

[24] Olzmann JA Carvalho P. Dynamics and functions of lipid droplets. Nat Rev Mol Cell Biol. (2019) 20:137–55. 10.1038/s41580-018-0085-z 30523332 PMC6746329

[25] Maitra U Li L. Molecular mechanisms responsible for the reduced expression of cholesterol transporters from macrophages by low-dose endotoxin. Arterioscler Thromb Vasc Biol. (2013) 33:24–33. 10.1161/ATVBAHA.112.300049 23117655 PMC3545450

[26] Liu X Wang N Fan SJ Zheng XC Yang YJ Zhu YF The citrus flavonoid naringenin confers protection in a murine endotoxaemia model through AMPK-ATF3-dependent negative regulation of the TLR4 signalling pathway. Sci Rep. (2016) 6:39735 10.1038/srep39735 28004841 PMC5177915

[27] Kapellos TS Taylor L Lee H Cowley SA James WS Iqbal AJ. A novel real time imaging platform to quantify macrophage phagocytosis. Biochem Pharmacol. (2016) 116:107–19. 10.1016/j.bcp.2016.07.011 27475716 PMC5012892

[28] Wu B Ni H Li J Zhuang X Zhang J Qi Z. The impact of circulating mitochondrial DNA on cardiomyocyte apoptosis and myocardial injury after tlr4 activation in experimental autoimmune myocarditis. Cell Physiol Biochem. (2017) 42:713–28. 10.1159/000477889 28618428

[29] Kean LS Grant AM Angeletti C Mahe Y Kuchler K Fuller RS. Plasma membrane translocation of fluorescent-labeled phosphatidylethanolamine is controlled by transcription regulators, PDR1 and PDR3. J Cell Biol. (1997) 138:255–70. 10.1083/jcb.138.2.255 9230069 PMC2138184

[30] Stevens HC Malone L Nichols JW. The putative aminophospholipid translocases, DNF1 and DNF2, are not required for 7-nitrobenz-2-oxa-1, 3-diazol-4-yl-phosphatidylserine flip across the plasma membrane of *Saccharomyces cerevisiae*. J Biol Chem. (2008) 283:35060–9. 10.1074/jbc.M802379200 18931395 PMC3259875

[31] Kasahara T Tomita K Murano H Harada T Tsubakimoto K Ogihara T. Establishment of an in vitro high-throughput screening assay for detecting phospholipidosis-inducing potential. Toxicol Sci. (2006) 90:133–41. 10.1093/toxsci/kfj067 16338956

[32] Yuan M Breitkopf SB Yang X Asara JM. A positive/negative ion-switching, targeted mass spectrometry-based metabolomics platform for bodily fluids, cells, and fresh and fixed tissue. Nat Protoc. (2012) 7:872–81. 10.1038/nprot.2012.024 22498707 PMC3685491

[33] Ivanova PT Milne SB Brown HA. Identification of atypical ether-linked glycerophospholipid species in macrophages by mass spectrometry. J Lipid Res. (2010) 51:1581–90. 10.1194/jlr.D003715 19965583 PMC3035522

[34] Zhao L Wan L Qiu X Li R Liu S Wang D A metabonomics profiling study on phlegm syndrome and blood-stasis syndrome in coronary heart disease patients using liquid chromatography/quadrupole time-of-flight mass spectrometry. Evid Based Compl Altern Med. (2014) 2014:385102 10.1155/2014/385102 PMC4129150 25140185

[35] del Fresno C Garcia-Rio F Gomez-Pina V Soares-Schanoski A Fernandez-Ruiz I Jurado T. Potent phagocytic activity with impaired antigen presentation identifying lipopolysaccharide-tolerant human monocytes: demonstration in isolated monocytes from cystic fibrosis patients. J Immunol. (2009) 182:6494–507. 10.4049/jimmunol.0803350 19414804

[36] Zhu X Huang G Jin P. Clinicopathological and prognostic significance of aberrant G protein-couple receptor 110 (GPR110) expression in gastric cancer. Pathol Res Pract. (2019) 215:539–45. 10.1016/j.prp.2018.12.004 30638950

[37] Zhang CP Wang Y Wang F Wang ZX Lu Y Xu Y. Quantitative profiling of glycerophospholipids during mouse and human macrophage differentiation using targeted mass spectrometry. Sci Rep. (2017) 7:412. 10.38/s41598-017-00341-2 28341849 PMC5428309

[38] Wallner S Orso E Grandl M Konovalova T Liebisch G Schmitz G. Phosphatidylcholine and phosphatidylethanolamine plasmalogens in lipid loaded human macrophages. PLoS ONE. (2018) 13:e0205706. 10.1371/journal.pone.0205706 30308051 PMC6181407

[39] Dawaliby R Trubbia C Delporte C Noyon C Ruysschaert JM Van Antwerpen P. Phosphatidylethanolamine is a key regulator of membrane fluidity in eukaryotic cells. J Biol Chem. (2016) 291:3658–67. 10.1074/jbc.M115.706523 26663081 PMC4751403

[40] Hardie DG. AMP-activated protein kinase-an energy sensor that regulates all aspects of cell function. Genes Dev. (2011) 25:1895–908. 10.1101/gad.17420111 21937710 PMC3185962

[41] Wu G Zhang L Li T Zuniga A Lopaschuk GD Li L. Choline supplementation promotes hepatic insulin resistance in phosphatidylethanolamine N-methyltransferase-deficient mice via increased glucagon action. J Biol Chem. (2013) 288:837–47. 10.1074/jbc.M112.415117 23179947 PMC3543033

[42] Jacobs RL Lingrell S Dyck JR Vance DE. Inhibition of hepatic phosphatidylcholine synthesis by 5-aminoimidazole-4-carboxamide-1-β-4-ribofuranoside is independent of AMP-activated protein kinase activation. J Biol Chem. (2007) 282:4516–23. 10.1074/jbc.M605702200 17179149

[43] Chu ZT Tsuchiya N Kyogoku C Ohashi J Qian YP Xu SB. Association of Fcgamma receptor IIb polymorphism with susceptibility to systemic lupus erythematosus in Chinese: a common susceptibility gene in the Asian populations. Tissue Antigens. (2004) 63:21–7. 10.1111/j.1399-0039.2004.00142.x 14651519

[44] Remmerie A Scott CL. Macrophages and lipid metabolism. Cell Immunol. (2018) 330:27–42. 10.1016/j.cellimm.2018.01.020 29429624 PMC6108423

[45] Green C Mitchell S Speakman J. Energy balance and the sphingosine-1-phosphate/ceramide axis. Aging. (2017) 9:2463–4. 10.18632/aging.101347 29242408 PMC5764382

[46] Cuschieri J Billigren J Maier RV. Endotoxin tolerance attenuates LPS-induced TLR4 mobilization to lipid rafts: a condition reversed by PKC activation. J Leukoc Biol. (2006) 80:1289–97. 10.1189/jlb.0106053 16959900

[47] de la Haba C Morros A Martinez P Palacio JR. LPS-induced macrophage activation and plasma membrane fluidity changes are inhibited under oxidative stress. J Membr Biol. (2016) 249:789–800. 10.1007/s00232-016-9927-9 27619206

[48] Vance DE Li Z Jacobs RL. Hepatic phosphatidylethanolamine N-methyltransferase, unexpected roles in animal biochemistry and physiology. J Biol Chem. (2007) 282:33237–41. 10.1074/jbc.R700028200 17881348

[49] Vance DE. Physiological roles of phosphatidylethanolamine N-methyltransferase. Biochim Biophys Acta. (2013) 1831:626–32. 10.1016/j.bbalip.2012.07.017 22877991

[50] Ireland R Schwarz B Nardone G Wehrly TD Broeckling CD Chiramel AI. Unique francisella phosphatidylethanolamine acts as a potent anti-inflammatory lipid. J Innate Immun. (2018) 10:291–305. 10.1159/000489504 29969788 PMC6757151

[51] Hoogendijk AJ Pinhancos SS van der Poll T Wieland CW. AMP-activated protein kinase activation by 5-aminoimidazole-4-carbox-amide-1-beta-D-ribofuranoside (AICAR) reduces lipoteichoic acid-induced lung inflammation. J Biol Chem. (2013) 288:7047–52. 10.1074/jbc.M112.413138 23322781 PMC3591614

[52] Boss M Newbatt Y Gupta S Collins I Brune B Namgaladze D. AMPK-independent inhibition of human macrophage ER stress response by AICAR. Sci Rep. (2016) 6:32111. 10.1038/srep32111 27562249 PMC4999824

[53] Garcia D Shaw RJ. AMPK: mechanisms of cellular energy sensing and restoration of metabolic balance. Mol Cell. (2017) 66:789–800. 10.1016/j.molcel.2017.05.032 28622524 PMC5553560

[54] Wang Q Liu S Zhai A Zhang B Tian G. AMPK-mediated regulation of lipid metabolism by phosphorylation. Biol Pharm Bull. (2018) 41:985–93. 10.1248/bpb.b17-00724 29709897

[55] Nguyen TB Louie SM Daniele JR Tran Q Dillin A Zoncu R. DGAT1-dependent lipid droplet biogenesis protects mitochondrial function during starvation-induced autophagy. Dev Cell. (2017) 42:9–21 e25. 10.1016/j.devcel.2017.06.003 28697336 PMC5553613

[56] Carling D Thornton C Woods A Sanders MJ. AMP-activated protein kinase: new regulation, new roles? Biochem J. (2012) 445:11–27. 10.1042/BJ20120546 22702974

[57] Herzig S Shaw RJ. AMPK: guardian of metabolism and mitochondrial homeostasis. Nat Rev Mol Cell Biol. (2018) 19:121–35. 10.1038/nrm.2017.95 28974774 PMC5780224

[58] Hu K Gong X Ai Q Lin L Dai J Cai L. Endogenous AMPK acts as a detrimental factor in fulminant hepatitis via potentiating JNK-dependent hepatocyte apoptosis. Cell Death Dis. (2017) 8:e2637. 10.1038/cddis.2017.62 28252653 PMC5386558

[59] Chiang C-F Chao T-T Su Y-F Hsu C-C Chien C-Y Chiu K-C. Metformin-treated cancer cells modulate macrophage polarization through AMPK-NF-_κ_B signaling. Oncotarget. (2017) 8:20706–18. 10.18632/oncotarget.14982 28157701 PMC5400538

[60] Daval M Foufelle F Ferre P. Functions of AMP-activated protein kinase in adipose tissue. J Physiol. (2006) 574(Pt 1):55–62. 10.1113/jphysiol.2006.111484 16709632 PMC1817807

[61] Seeley JJ Ghosh S. Molecular mechanisms of innate memory and tolerance to LPS. J Leukoc Biol. (2017) 101:107–19. 10.1189/jlb.3MR0316-118RR 27780875

